# Vitamin C for preventing atrial fibrillation in high risk patients: a systematic review and meta-analysis

**DOI:** 10.1186/s12872-017-0478-5

**Published:** 2017-02-01

**Authors:** Harri Hemilä, Timo Suonsyrjä

**Affiliations:** 1 Department of Public Health, POB 20, University of Helsinki, Tukholmankatu 8 B 2B, FI-00014 Helsinki, Finland; 2 Emergency Clinic, Helsinki University Central Hospital, Meilahti Hospital, Helsinki, Finland

**Keywords:** Ascorbic acid, Arrhythmia, Antioxidant, Atrial fibrillation, Cardiac surgery, Cardioversion, Intensive care

## Abstract

**Background:**

Atrial fibrillation (AF), a common arrhythmia contributing substantially to cardiac morbidity, is associated with oxidative stress and, being an antioxidant, vitamin C might influence it.

**Methods:**

We searched the Cochrane CENTRAL Register, MEDLINE, and Scopus databases for randomised trials on vitamin C that measured AF as an outcome in high risk patients. The two authors independently assessed the trials for inclusion, assessed the risk of bias, and extracted data. We pooled selected trials using the Mantel-Haenszel method for the risk ratio (RR) and the inverse variance weighting for the effects on continuous outcomes.

**Results:**

We identified 15 trials about preventing AF in high-risk patients, with 2050 subjects. Fourteen trials examined post-operative AF (POAF) in cardiac surgery patients and one examined the recurrence of AF in cardioversion patients. Five trials were carried out in the USA, five in Iran, three in Greece, one in Slovenia and one in Russia.

There was significant heterogeneity in the effect of vitamin C in preventing AF. In 5 trials carried out in the USA, vitamin C did not prevent POAF with RR = 1.04 (95% CI: 0.86–1.27). In nine POAF trials conducted outside of the USA, vitamin C decreased its incidence with RR = 0.56 (95% CI: 0.47–0.67). In the single cardioversion trial carried out in Greece, vitamin C decreased the risk of AF recurrence by RR = 0.13 (95% CI: 0.02–0.92).

In the non-US cardiac surgery trials, vitamin C decreased the length of hospital stay by 12.6% (95% CI 8.4–16.8%) and intensive care unit (ICU) stay by 8.0% (95% CI 3.0–13.0%). The US trials found no effect on hospital stay and ICU stay. No adverse effects from vitamin C were reported in the 15 trials.

**Conclusions:**

Our meta-analysis indicates that vitamin C may prevent post-operative atrial fibrillation in some countries outside of the USA, and it may also shorten the duration of hospital stay and ICU stay of cardiac surgery patients. Vitamin C is an essential nutrient that is safe and inexpensive. Further research is needed to determine the optimal dosage protocol and to identify the patient groups that benefit the most.

**Electronic supplementary material:**

The online version of this article (doi:10.1186/s12872-017-0478-5) contains supplementary material, which is available to authorized users.

## Background

Atrial fibrillation (AF) is a common cardiac rhythm disturbance which can lead to severe consequences such as stroke [1]. AF can be triggered by various stressful conditions; for example, about 30% of patients undergoing cardiac operations, such as coronary artery bypass grafting (CABG), suffer from post-operative AF (POAF) [2]. AF is associated with oxidative stress and it seems that the cause-effect relation may work in both directions. In animal studies, tachycardia increased the levels of oxidative stress markers [3, 4], while oxidative stress increased the susceptibility of isolated hearts to tachycardia [5]. Since several ion channels which are expressed in the atria are sensitive to the redox state, oxidative stress and antioxidants might influence the electrophysiology of the atria [6].

Vitamin C is a water soluble antioxidant which may protect against oxidative stress. In some studies, cardiac surgery decreased vitamin C levels consistent with increased oxidative stress [7–9]. There is also evidence that vitamin C may have a treatment effect on some cardiovascular disorders. In patients undergoing cardiac operations, vitamin C increased cardiac perfusion after surgery [10], and decreased the level of creatine kinase MB [11, 12]. A meta-analysis of patients with atherosclerosis or heart failure found that vitamin C improved endothelial function [13], and another found that vitamin C reduced blood pressure [14]. In addition, a recent meta-analysis found that vitamin C decreased the risk of contrast-induced acute kidney injury in patients undergoing coronary angiography [15].

In 2001, Carnes et al. reported that vitamin C administration seemed to prevent POAF, but they used historical controls instead of concurrent randomized controls [3]. Nevertheless, that study led to a series of randomized trials on vitamin C against POAF.

AF is a common cardiac arrhythmia and vitamin C is a safe and inexpensive essential nutrient. Approximately 0.01 g/day of vitamin C prevents scurvy but the safe dose range extends to grams per day [16, 17]. The possibility that vitamin C might have preventive effects against AF, even in restricted population groups, is worth examination. The goal of this systematic review was to analyse the preventive effect of vitamin C against AF in patients with a high risk of AF.

## Methods

### Selection criteria for trials and the searches

**Fig. 1:**
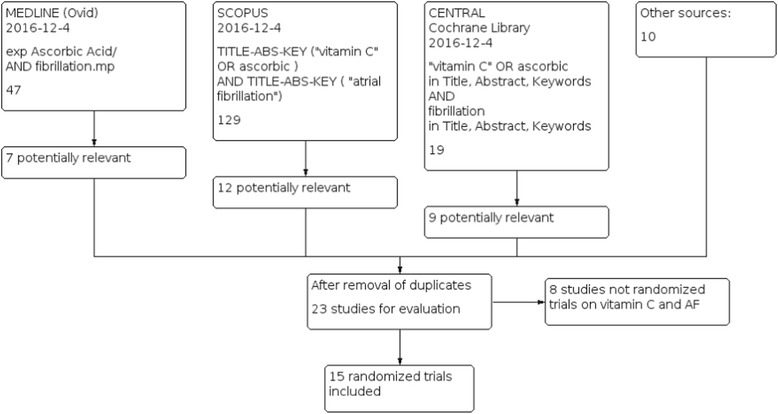
Flow diagram of the searches. The search terms and the number of identified records are shown in this figure

We selected randomized controlled vitamin C trials which measured the occurrence of AF in patients at a high risk of AF. We considered high risk patients as those undergoing cardiac surgery and those going to cardioversion for whom there is a high risk of recurrence of AF. The use of placebo in the control group was not required. We did not set limits on the dose or duration of vitamin C administration. We searched the Cochrane Register (CENTRAL; Cochrane Library), MEDLINE (Ovid) and Scopus (Ovid). We searched all databases from their inception to December 4, 2016, and we imposed no restriction on the language of publication. The search strategies are detailed in Fig. 1. We also searched ClinicalTrials.gov and the WHO International Clinical Trials Registry Platform (ICTRP) under “vitamin C” and “fibrillation”. We checked reference lists of the trials included and relevant reviews, and contacted the authors of published trials to ask if they knew of ongoing or unpublished trials. We included trials reported as full-text or abstract, and also unpublished trials. We identified 15 trials in all [18–32] (Fig. 1; Table 1; Additional file 1).

**Table 1:** Characteristics of included trials

Trial (ref.)	Country	Setting	No. of Participants	Mean age (yr)	Proportion males
van Wagoner 2003 [27]	USA	CABG	346	63	NA
Donovan 2012 [30]	USA	CABG or valvular surgery	304	64	76%
Sadeghpour 2015 [31]	Iran	CABG or valvular surgery	290	56	66%
Bjordahl 2012 [18]	USA	CABG	185	63	67%
Papoulidis 2011 [21]	Greece	CABG	170	73	71%
Sarzaeem 2014 [26]	Iran	CABG	170	59	69%
Samadikhah 2014 [25]	Iran	CABG	120	61	68%
Antonic 2016 [23]	Slovenia	CABG	105	64	78%
Dehghani 2014 [19]	Iran	CABG	100	61	74%
Eslami 2007 [20]	Iran	CABG	100	60	67%
Korantzopoulos 2005 [32]	Greece	Cardioversion	44	68	59%
Rebrova 2012 [24]	Russia	CABG	40	59	100%
Healy 2010 [29]	USA	CABG or valvular surgery	30	NA	NA
Colby 2011 [28]	USA	CABG or valvular surgery	24	65	79%
Polymeropoulos 2015 [22]	Greece	CABG	22	70	59%

The trials are listed by the number of patients. The two largest trials [27, 30], both carried out at the USA, have not been published because of their negative findings. For the US trials, the weighted mean age was 63.4 years and for the non-US trials, it was 61.8 years. For the US trials, the overall proportion of males was 73%, and for the non-US trials, it was 70%

*CABG* Coronary artery bypass grafting, *NA* Not available

### Outcomes

Our primary outcome was the occurrence of AF. As secondary outcomes, we analysed the length of hospital stay and the length of intensive care unit (ICU) stay.

### Selection of studies and data extraction

The two authors independently screened the titles and abstracts, and identified trials for inclusion. One author (HH) extracted study characteristics and outcomes from the trials and entered the data to the Review Manager 5 (RevMan) program [33]. Both authors checked the data entered in the RevMan program against the original trial reports. We contacted all authors to ask for more details (see Additional file 1).

### Quality assessment of the trials

Both authors assessed the quality of included trials using the Cochrane Collaboration’s tool for assessing the risk of bias, our assessments being shown in the Additional file 1. We assessed the trials for the following criteria: random sequence generation, allocation concealment, blinding of participants and personnel, blinding of outcome assessment, incomplete outcome data, selective reporting, and other bias. We gave each item a designation of high, low, or unclear risk of bias.

### Statistical methods

We analysed dichotomous data on the incidence of AF as risk ratios (RR). Our main analysis of continuous data was as percentage effects, since that adjusts for baseline variations between trials. For the length of hospital stay, we also calculated the effect of vitamin C on the number of days the patient stayed at the hospital.

Some studies used the Mann–Whitney test in the calculation of the P-values since, for skewed data such as the length of hospital stay, it is preferable to the *t*-test. In some trials, the Mann–Whitney P-values calculated by the original authors were incompatible with the reported SD values. Therefore, in 3 studies we adjusted the SD values for hospital stay [21, 23, 31], and in 2 studies we adjusted the SD values for ICU stay [21, 23], to make them consistent with the Mann–Whitney P-values; see the Additional file 1.

In the Korantzopoulos trial [32], 1 cell in the 2 × 2 table had only 1 case of AF recurrence. We therefore calculated the P-value by the mid-P modification of the Fisher exact test [34].

We pooled the selected trials with the RevMan program, using the Mantel-Haenszel option for RRs and the inverse variance option for continuous outcomes, with the fixed effect option for both. We used the Chi^2^ test and the I^2^ statistic to assess statistical heterogeneity among the trials in each meta-analysis [35]. A value of I^2^ greater than about 70% indicates a high level of heterogeneity. We used 2-tailed P values in this review.

## Results

### Description of the trials

We identified 15 trials which examined the effect of vitamin C on preventing AF in patients at a high risk of AF [18–32]. See the flow diagram of the searches in Fig. 1. Fourteen trials examined patients undergoing cardiac surgery, either CABG [18–27] or CABG and valvular surgery [28–31]. The 15th trial examined the recurrence of AF after a successful cardioversion [32] (Table 1).

Five trials were carried out in the USA [18, 27–30], 5 in Iran [19, 20, 25, 26, 31], 3 in Greece [21, 22, 32], 1 in Slovenia [23], and 1 in Russia [24]. The total number of participants was 2050. The mean age ranged from 56 to 73 years, and the proportion of males varied from 59 to 100% (median 69%) in the trials that reported sex distribution (Table 1).

Most POAF trials administered 2 g of vitamin C within about 12 h before the operation and 1–2 g/day for 5 days after the operation, and followed patients for the occurrence of AF for the same period. In most trials, vitamin C was administered as tablets, although 5 trials administered it intravenously [21–23, 26, 31]. See the description of dosages in the Additional file 1.

In the cardioversion trial [32], 2 g vitamin C was administered before the cardioversion and thereafter 1 g/day of vitamin C for 7 days. After a successful cardioversion, participants were followed for 7 days for the recurrence of AF.

Because of the selection criteria, all the trials were randomized. Korantzopoulos et al. [32] mentioned that the cardioversion patients bought vitamin C tablets themselves and thus knew the treatment, but the physician who was responsible for cardioversion and follow-up was unaware of the treatment. A placebo was not used in 6 POAF trials [19, 20, 23, 24, 29, 32], but in other trials participants were administered a placebo. Four trials [23, 24, 29, 32] did not report that the physicians in charge of treatments and assessment of outcome were blinded, but in other trials that was the case. None of the trials had substantial or unbalanced drop-out rates. See the Additional file 1 for the details of the methods.

### Effect of vitamin C on atrial fibrillation

**Fig. 2:**
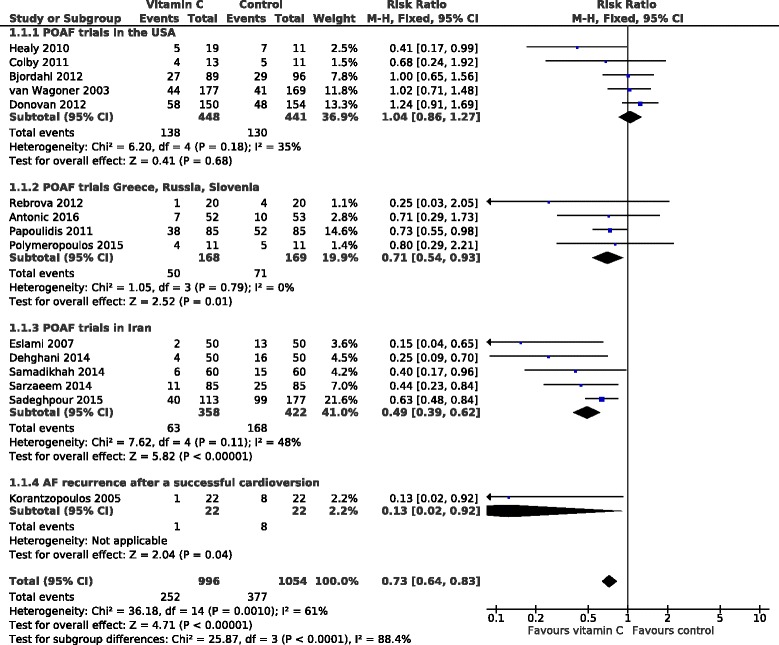
Effect of vitamin C on the occurrence of AF in high risk patients. The upper three subgroups are trials on POAF and the lowest subgroup includes the cardioversion study. The horizontal lines indicate the 95% CI for the effect of vitamin C and the square in the middle of the horizontal line indicates the point estimate of the effect in the particular trial. The diamond shapes indicate the pooled effects on the symptoms and its 95% CI

Figure 2 shows the effect of vitamin C on the occurrence of AF in high risk patients. Over all the 15 trials, vitamin C decreased the risk of AF by 27%. However, there is significant heterogeneity between the 15 trials with I^2^ = 61% (*P* = 0.001 in the heterogeneity test), which indicates that no single estimate of effect, such as the 27% mean effect, is consistent with the results of all the 15 trials, and thus the causes of the heterogeneity should be explored.

Because of the variations in the clinical context and in the results, we divided all the trials into 4 subgroups. The 14 trials on POAF were divided into trials carried out a) in the USA, b) in Greece, Slovenia, and Russia, and c) in Iran, and the 15th trial is included as d) the cardioversion trial in Greece (Fig. 2). There is very strong evidence that the estimates calculated for these 4 subgroups are heterogeneous with I^2^ = 88% (*P* = 10^−5^). The high level of heterogeneity is caused by the US trials, which found no effect of vitamin C. If the 5 US trials are removed, the remaining 10 non-US trials are not heterogeneous, I^2^ = 38% (*P* = 0.10), and the pooled estimate indicates a 45% decrease (95% CI 35 to 54%; *P* = 10^−10^) in the occurrence of AF. If we further remove 4 non-US trials that had some concerns about randomization or blinding [23, 24, 31, 32], the effect estimate remains essentially the same indicating a 48% (95% CI 34 to 60%) decrease in the incidence of AF (see Additional file 2). Thus, there is very strong evidence from the 10 non-US trials that vitamin C decreases the risk of AF in high risk patients.

**Fig. 3:**
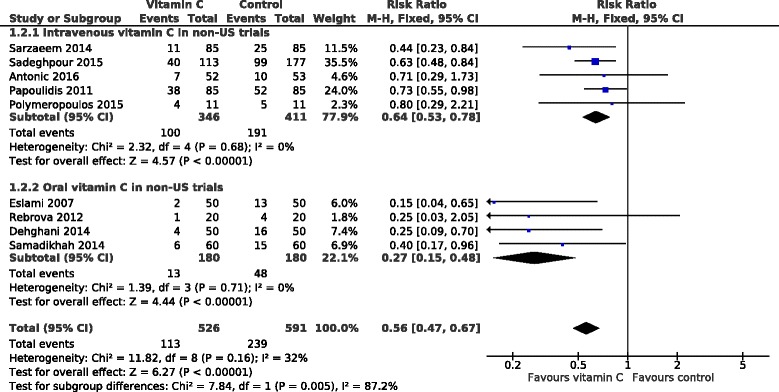
Effect of vitamin C on the occurrence of POAF in non-US trials by vitamin C administration method. The trials in the upper subgroup administered vitamin C intravenously and the trials in the lower subgroup administered it orally. The horizontal lines indicate the 95% CI for the vitamin C effect and the square in the middle of the horizontal line indicates the point estimate of the effect in the particular trial. The diamond shapes indicate the pooled effects on the symptoms and its 95% CI

The 5 POAF trials in Iran found a 51% decrease in the incidence of AF and the 4 POAF trials in Greece, Slovenia, and Russia found a 29% decrease. When these 9 trials were pooled to a group of non-US POAF trials, vitamin C decreased the incidence of AF by 44% (33 to 53%) (Fig. 3).

In a direct comparison of the 5 US POAF trials against the 5 Iran POAF trials, there is very strong evidence of heterogeneity with I^2^ = 95% (*P* = 10^−5^) (Additional file 2). Thus, vitamin C seems to have significantly different effect in these two countries.

Korantzopoulos et al. studied the recurrence of AF within 1 week after a successful cardioversion, finding that vitamin C decreased the rate of AF recurrence by 87% (*P* = 0.012).

All the POAF trials administered 1–2 g/day of vitamin C. Most trials gave it as tablets, whereas 5 gave it intravenously. These 2 methods of administration lead to different vitamin C levels in the body and we compared the 2 methods among the non-US POAF trials in Fig. 3. Oral administration decreased the occurrence of POAF by 73% and intravenous administration by 36%. There is strong evidence of heterogeneity between the 2 administration methods with I^2^ = 87% (*P* = 0.005).

### Effect of vitamin C on hospital stay

**Fig. 4:**
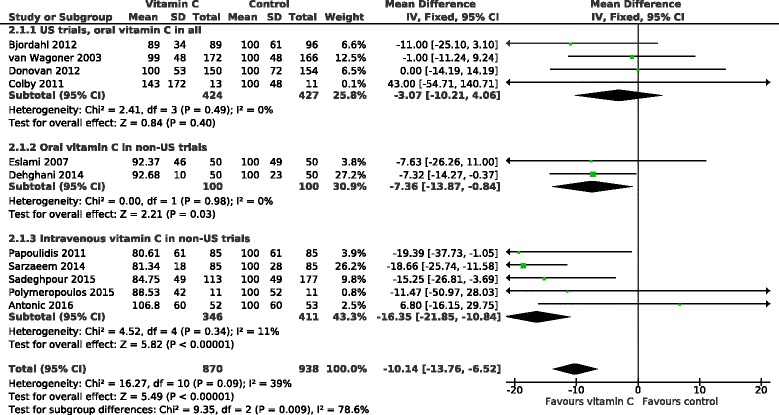
Effect of vitamin C on the length of hospital stay in percentages. The subgroup on the top includes the US trials, all of which administered vitamin C orally. The non-US trials are divided into groups by oral and intravenous vitamin C administration. The durations of hospital stay were transformed to the relative scale; thus the duration in the corresponding placebo group was given a value of 100%. Thereby the difference between the vitamin C and control groups gives the effect of vitamin C directly as a percentage. The horizontal lines indicate the 95% CI for the vitamin C effect and the square in the middle of the horizontal line indicates the point estimate of the effect in the particular trial. The diamond shapes indicate the pooled effects on the symptoms and their 95% CI

The effect of vitamin C on the hospital stay in 11 POAF trials is shown in Fig. 4. On average, vitamin C shortened the hospital stay by 10.1%. Because of the heterogeneity found in the effects of vitamin C against POAF, we divided the trials into the US and the non-US trials also in this analysis. The US trials found no effect on hospital stay whereas in the non-US cardiac surgery trials, vitamin C decreased the length of hospital stay by 12.6% (95% CI 8.4 to 16.8%; *P* = 10^−8^). Only 1 of the non-US trials had some concerns about blinding [23], and its exclusion had only a small effect on the pooled effect estimate indicating a 13.3% (9.0 to 17.6%) decrease in the length of hospital stay (Additional file 2).

The non-US trials were further divided into oral and intravenous trials. There is strong evidence of heterogeneity between the 3 subgroups with I^2^ = 78% (*P* = 0.009) (Fig. 4). In the non-US trials, intravenous administration shortened the length of hospital stay by 16% and oral administration by 7% and there was evidence of heterogeneity between the oral and intravenous non-US trials with I^2^ = 76% (*P* = 0.039).

**Fig. 5:**
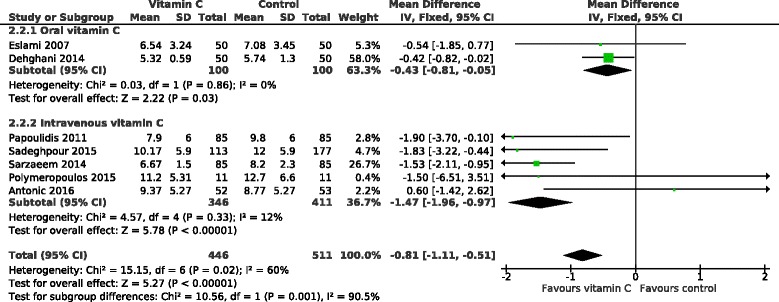
Effect of vitamin C on the length of hospital stay in days. The non-US trials are divided into groups by oral and intravenous vitamin C administration. The US trials are not shown since they found no effect of vitamin C on hospital stay (Fig. 4). The horizontal lines indicate the 95% CI for the vitamin C effect and the square in the middle of the horizontal line indicates the point estimate of the effect in the particular trial. The diamond shapes indicate the pooled effects on the symptoms and their 95% CI

The relative effect, i.e., the effect in percentages, adjusts for baseline variations in the patient groups and is therefore an informative effect measure when pooling trial results in Fig. 4. Nevertheless, since the effect on hospital stay as actual days has more direct practical impact, we also calculated the effect of vitamin C on the days in hospital in Fig. 5. In the non-US trials, intravenous vitamin C shortened hospital stay on average by 1.47 days and oral vitamin C by 0.43 days.

**Fig. 6:**
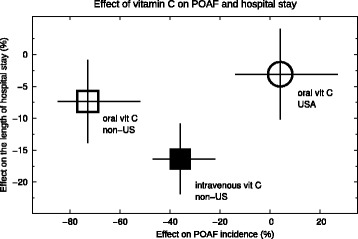
Effect of oral and intravenous vitamin C on POAF and on hospital stay. The open square indicates the pooled effect of oral vitamin C in the non-US trials and the filled in square indicates the pooled effect of intravenous vitamin C trials in the non-US trials. The open circle indicates the pooled effect of oral vitamin C in the US trials. The estimates are from Figs. 2, 3 and 4. The horizontal and vertical lines indicate the 95% CI ranges for each effect. The Rebrova (2012) [24] and Samadikhah (2014) [25] trials contribute to the estimate of oral vitamin C effect on POAF occurrence in the non-US trials, but they did not report the findings on hospital stay

Since oral and intravenous vitamin C differed in their effects on the occurrence of POAF and on the duration of hospital stay, we plotted the estimates of effect in Fig. 6. In the non-US trials, oral vitamin led to a greater effect on POAF occurrence but to a lesser effect on hospital stay compared with intravenous vitamin C. The US trials found no benefit on either outcome.

### Effect of vitamin C on ICU stay

**Fig. 7:**
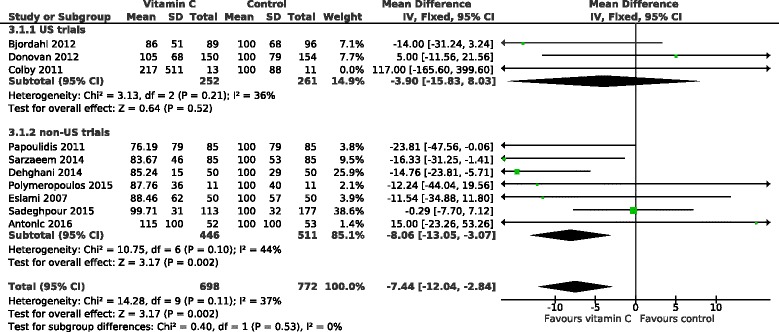
Effect of vitamin C on the length of ICU stay. The US and non-US trials are separated in this figure. The durations of ICU stay were transformed to the relative scale; thus the duration in the corresponding placebo group was given a value of 100%. Thereby the difference between the vitamin C and control groups gives the effect of vitamin C directly as a percentage. The horizontal lines indicate the 95% CI for the vitamin C effect and the square in the middle of the horizontal line indicates the point estimate of the effect in the particular trial. The diamond shapes indicate the pooled effects on the symptoms and their 95% CI

The effect of vitamin C on the length of ICU stay in the POAF trials is shown in Fig. 7. Vitamin C shortened ICU stay by 8% (*P* = 0.002) in the 7 non-US trials and there is no evidence of heterogeneity between the non-US trials (*P* = 0.1). Three US trials found no effect.

## Discussion

Our systematic review was formulated as an examination of the effects of vitamin C on the occurrence of AF in people at a high risk for AF. Since patients undergoing cardiac surgery and cardioversion may suffer from acute oxidative stress, vitamin C administration might have an influence in such special conditions. We found 15 randomized trials on vitamin C for preventing AF in high risk patients. On average, vitamin C decreased the occurrence of AF by 27%, but there was significant heterogeneity in the results. We also found a 10% decrease in hospital stay in cardiac surgery patients. Heterogeneity indicates that a single average estimate of effect cannot be valid for all of the 15 trials and justified exploration to identify the causes of this heterogeneity.

Five of the trials included in Fig. 2 were carried out in Iran and the pooled estimate indicates a 51% reduction in POAF incidence with vitamin C administration. In contrast, 5 trials in the USA found that vitamin C produced no benefit. These 2 sets of trials are significantly incompatible. We do not consider that methodological differences between the US and Iran trials are the most likely explanations for the divergence between these 2 sets of trials. Previous research has pointed out that treatment effects can differ between less and more developed countries. Panagiotou et al. [36] found several cases in which trials in less developed countries showed more favourable treatment effects than trials in more developed countries. Although methodological variations may explain some of the differences, it is also likely that there are genuine differences between many treatment effects between substantially different cultures. Wealth is strongly correlated with life-style factors including nutrition, and with differences in hospital treatments. Such differences might explain the divergence between the results in the 5 US and the 5 Iran POAF trials.

Among the non-US trials, oral vitamin C had a greater effect on POAF occurrence than intravenous vitamin C (Fig. 3). There is a substantial difference in the pharmacokinetics between oral and intravenous vitamin C, so that the same intravenous dose leads to much higher vitamin levels in plasma than the oral dose [16, 37]. Furthermore, intravenous vitamin C may be more reliable for postoperative patients since delayed gastric emptying is a frequent concern. Therefore the greater effect of oral vitamin C on POAF occurrence was against our expectation. On the other hand, the greater effect of intravenous vitamin C on the hospital stay is consistent with greater effects of higher plasma levels.

POAF has previously been correlated with a longer stay in hospital, but it is not known whether the longer hospital stay is caused by the episode of POAF or whether both of them are caused by other factors [38]. Among the non-US trials, oral vitamin C had a greater effect on POAF, but a smaller effect on hospital stay compared with intravenous vitamin C (Fig. 6). This negative correlation between the effects on POAF and on hospital stay conflicts with the notion that POAF is the cause of a longer hospital stay. The divergence we found in the effects of oral and intravenous vitamin C administration indicates that the 2 methods of vitamin C administration should be further studied by head-to-head comparisons in 3-arm randomized trials instead of just comparing independent trials that have various other differences simultaneously.

As the methodological inclusion criterion, we required that the trials be randomized. Six trials with cardiac surgery patients did not use an explicit placebo [19, 20, 23, 24, 29, 32]. However, other medications serve as a functional placebo to vitamin C in cardiac surgery trials. It seems highly unlikely that such patients might notice whether vitamin C is administered along with the other medications, so that the lack of an explicit placebo would substantially bias observations in the 5 trials. Three POAF trials [23, 24, 29] did not report that physicians in charge of treatments and outcome assessment were blinded for the trial groups, but in other trials they were blinded. In the Korantzopoulos et al. trial on the recurrence of AF after cardioversion, patients bought vitamin C tablets themselves and knew their treatment [32]. However, a large meta-analysis showed that placebo has minimal or no effects on binary outcomes [39], such as the occurrence of AF.

In sensitivity analyses, we excluded trials that had some concerns about possible bias in the comparison. However, the estimates of vitamin C effects were not changed. In addition, the heterogeneity between the US and the non-US trials was not influenced by the exclusion of trials that had some concerns about possible bias.

Two relatively large US trials on POAF were not published because their results were negative [27, 30]. Nevertheless, we do not consider that publication bias is a likely explanation for our main findings. The evidence of the benefit of vitamin C in the non-US trials is very strong and there should be a particularly large number of unpublished non-US trials to explain the findings purely as a result of researchers publishing just the positive findings. In particular, publication bias is not a reasonable explanation for the significant difference between oral and intravenous administration.

In the first study on vitamin C for preventing POAF, Carnes et al. described their protocol as follows: “patients scheduled for primary CABG surgery were given 2 g ascorbic acid (extended release) the night before surgery, followed by 500-mg doses twice daily for the 5 days after surgery” [3]. All subsequent POAF trials used dosage protocols that are only minor modifications of the Carnes method. Although the first question after the publication of a positive result should be whether the result can be repeated, subsequent trials should also investigate protocol variations to determine the optimal protocol.

Vitamin C is water soluble, and its concentration in plasma increases within 1–2 h of oral administration and decreases thereafter [16, 37]. On the basis of such pharmacokinetics, it seems unlikely that several days administration before the cardiac operation might lead to further benefits. In 2 POAF trials, vitamin C was administered intravenously 3 h before the operation [21] or immediately before [31] and both produced a significant benefit. Longer administration before the operation might not be needed.

Another time-dependent question is about the length of vitamin C administration after the operation. In the POAF trials, vitamin C administration was continued for 5 days after the operation and the cardioversion trial continued administration for 7 days. Assuming that the greatest peak of oxidative stress occurs during and soon after the operation or cardioversion, a shorter administration period might be sufficient. In a trial with severe burn patients, vitamin C was administered intravenously for only 24 h after hospitalization but the dose was particularly high with 66 mg/kg/h (i.e., 110 g per 70 kg per 24 h) [40]. Compared with the control group, the level of vitamin C remained much higher in the vitamin groups for 3 days. In the vitamin C group, the length of mechanical ventilation was 43% shorter (*P* = 0.03) and there was a significant decrease in the requirement for infusion fluids [40], indicating that such a 1-day administration at a particularly high dose level might also be effective. Accordingly, the dose and the length of vitamin C administration should also be investigated in further trials.

## Conclusions

Trials carried out in Iran and Greece indicate that vitamin C may prevent AF after cardiac surgery or cardioversion. However, trials with cardiac surgery patients carried out in the USA found no benefit. Thus, trials in less wealthy countries should be carried out to optimize the protocol for vitamin C administration and to determine which patient groups get the most benefit, for example, by examining vitamin C status before the cardiac operation or cardioversion. Further trials in wealthy countries may investigate the effect of vitamin C against AF in patients who have a particularly low documented level of vitamin C, but there seems to be no rationale to study unselected cardiac surgery patients further.

## Additional files

Additional file 1: Description of the included trials and the risk of bias assessment. (PDF 203 kb)
Additional file 2: Additional forest plots showing the calculations. (PDF 957 kb)
