# Supplementary material for: Vitamin C for preventing atrial fibrillation in high risk patients: a systematic review and meta-analysis
Source: BMC Cardiovasc Disord. 2017 Feb 1;17:49. doi: 10.1186/s12872-017-0478-5 (PMC5286679; doi:10.1186/s12872-017-0478-5)
Supplement: Additional file 1: — Description of the included trials and the risk of bias assessment. (PDF 203 kb) [file 12872_2017_478_MOESM1_ESM.pdf]

Vitamin C for preventing atrial fibrillation in high risk patients: a systematic review and meta-analysis

Harri Hemilä and Timo Suonsyrjä

BMC Cardiovascular Disorders
https://bmccardiovascdisord.biomedcentral.com/

DOI 10.1186/s12872-017-0478-5
https://dx.doi.org/10.1186/s12872-017-0478-5

Additional file 1

version 2017-1-27

Harri Hemilä
Department of Public Health, POB 20
University of Helsinki
Tukholmankatu 8 B 2B
FI-00014 Helsinki
Finland

E-mail: harri.hemila@helsinki.fi
Home page: http://www.mv.helsinki.fi/home/hemila

Contents

Page

2 Risk of Bias assessment as a figure
3 Descriptions of the included randomized trials
37 Reasons for excluding 8 studies
38 References for excluded studies

	Random sequence generation (selection bias)	Allocation concealment (selection bias)	Blinding of participants and personnel (performance bias)	Blinding of outcome assessment (detection bias)	Incomplete outcome data (attrition bias)	Selective reporting (reporting bias)	Other bias
Antonic 2016	+	+	?	+	+	+	+
Bjordahl 2012	+	+	+	+	+	+	+
Colby 2011	+	+	+	+	+	+	+
Dehghani 2014	+	+	+	+	+	+	+
Donovan 2012	+	+	+	+	+	+	+
Eslami 2007	+	+	+	+	+	+	+
Healy 2010	+	?	?	?	?	-	?
Korantzopoulos 2005	+	+	?	+	+	+	+
Papoulidis 2011	+	+	+	+	+	+	+
Polymeropoulos 2015	+	+	+	+	+	+	+
Rebrova 2012	+	+	?	?	+	+	+
Sadeghpour 2015	?	+	+	+	+	+	+
Samadikhah 2014	+	+	+	+	+	-	+
Sarzaeem 2014	+	+	+	+	+	+	+
van Wagoner 2003	+	+	+	+	+	+	+

Antonic 2016

**Methods**	Randomized trial<br>http://dx.doi.org/10.1016/j.jjcc.2016.01.010<br>https://www.ncbi.nlm.nih.gov/pubmed/26917198
**Participants**	Slovenia, CABG patients, 82 M / 23 F; mean 64 yr (SD 12 yr); 52 vit C / 53 control.<br><br>**Inclusion:** Patients who were scheduled for an elective CABG.<br><br>**Exclusion:** Emergency operations, any concomitant valve or other surgery, preoperative history of AF, permanent pacemaker, hyperoxaluria or history of nephrolithiasis, and off-pump surgery.
**Interventions**	**Vit C before the operation:**<br>**Dose:** 2 g<br>**Method:** iv<br>**Timing:** "24 h and 2 h prior to surgery"<br>**Vit C after operation:**<br>**Dose:** 1 g/d<br>**Method:** iv<br>**Duration:** 5 d.<br><br>**Placebo** was not explicitly used, but other iv medications may serve as the placebo
**Outcomes**	POAF "AF or flutter lasting >10 min or the requirement for an urgent intervention due to AF of flutter (e.g. electroconversion) due to profound symptoms or hemodynamic instability",<br>Length of hospital stay,<br>Length of ICU stay
**Notes**	The authors reported the P-values for the difference in the length of hospital stay and the length of ICU stay based on Mann-Whitney test.<br><br>We calculated the SD value for our analyses to be consistent with the Mann-Whitney P-value.<br>Additional information was received by emails from Miha Antonic on 2016-12-2, see below.<br>The calculation of sample size in the trial was not correct, see:<br>http://dx.doi.org/10.1016/j.jjcc.2016.10.010<br>However, that problem does not challenge the internal validity of the Antonic trial.

Risk of bias table

Bias	Authors' judgement	Support for judgement
Random sequence generation (selection bias)	Low risk	"The enrolled patients were then randomly assigned to .." (text) and "For the randomization, we used the www.random.org online service" (Miha Antonic email 2016-12-1).
Allocation concealment (selection bias)	Low risk	"The randomization process was as follows: First, 52 As and 53 Bs were randomly arranged from 1 to 105" ... "the order of admittance of the patients to surgery was strictly in concordance with the Hospital's electronic waiting lists, which are under close surveilance of Ministry of Health and cannot be in any way manipulated" (Miha Antonic email 2016-12-1).
Blinding of participants and personnel (performance bias)	Unclear risk	"there was no blinding (and therefore use of placebo) in the study" (Miha Antonic email 2016-12-1).
Blinding of outcome assessment (detection bias)	Low risk	"The diagnosis of AF however was made on the basis of 24h telemetry recordings (for 7 days), which makes it very straightforward and objective" though "the physician who looked at the telemetry recordings knew which group the patient belonged to" (Miha Antonic email 2016-12-1).
Incomplete outcome data (attrition bias)	Low risk	"there were no dropouts" (Miha Antonic email 2016-12-1).
Selective reporting (reporting bias)	Low risk	
Other bias	Low risk	

Bjordahl 2012

**Methods**	Randomised placebo-controlled trial.<br>http://www.ncbi.nlm.nih.gov/pubmed/23022248<br>http://dx.doi.org/10.1016/j.amjsurg.2012.03.012
**Participants**	USA, CABG patients, 124 M / 61 F; mean 63 yr (SD 12 yr); 89 vit C / 96 placebo.<br><br>**Inclusion:** >18 yr who were scheduled to undergo CABG.<br><br>**Exclusion:** current AF, temporary or permanent pacemaker, life expectancy <1 month, emergency surgery precluding the initiation of study protocol the evening before surgery, current pregnancy.
**Interventions**	**Vit C before the operation:**<br>**Dose:** 2 g<br>**Method:** po<br>**Timing:** "evening before surgery"<br>**Vit C after operation:**<br>**Dose:** 2 g/d<br>**Method:** po<br>**Duration:** 5 d.<br><br>**Placebo:** identical placebo capsules at the same intervals; the inert substance for both treatment and placebo capsules was talc.
**Outcomes**	POAF "Postoperative AF or atrial flutter for 10 minutes" (p 863),<br>Length of hospital stay,<br>Length of ICU stay
**Notes**	We received no reply to our emails asking for more details.

Risk of bias table

Bias	Authors' judgement	Support for judgement
Random sequence generation (selection bias)	Low risk	"Enrolled participants were randomized to either ... The pharmacy department maintained the randomization list ..." (p 863).
Allocation concealment (selection bias)	Low risk	"The pharmacy department maintained the randomization list and assigned participants to the placebo and treatment arms of the study in a blinded fashion. Participants, clinicians, and evaluators were blinded to the treatment assignments and the blind was not broken until after data analyses were complete" (p 863).
Blinding of participants and personnel (performance bias)	Low risk	See above.
Blinding of outcome assessment (detection bias)	Low risk	See above.
Incomplete outcome data (attrition bias)	Low risk	13 participants were withdrawn from analysis because of surgery postponement/cancellation, presence of exclusion criteria at the time of enrollment etc.
Selective reporting (reporting bias)	Low risk	
Other bias	Low risk	"Both ascorbic acid and inert placebo capsules were prepared by a custom pharmacy" (p 863) indicates that the products were not commercial.

Colby 2011

**Methods**	Randomised placebo-controlled trial.<br>http://www.ncbi.nlm.nih.gov/pubmed/21856809<br>http://dx.doi.org/10.2146/ajhp100703
**Participants**	USA, CABG and valvular surgery patients, 19 M / 5 F, mean 65 yr (SD 9 yr), 13 vit C / 11 placebo.<br><br>**Inclusion:** >18 yr who were scheduled to undergo CABG, valvular surgery, or both.<br><br>**Exclusion:** excluded if they were pregnant or had a history of renal calculi.
**Interventions**	**Vit C before the operation:**<br>**Dose:** 2 g<br>**Method:** po<br>**Timing:** "night before surgery"<br>**Vit C after operation:**<br>**Dose:** 1 g/d<br>**Method:** po<br>**Duration:** 5 d.<br><br>**Placebo:** Both vit C and placebo were placed into identical capsules to allow for double-blinding.
**Outcomes**	POAF "Post-CTS AF, defined as any documented AF of more than five minutes' duration occurring between the day of surgery and postoperative day 4" (p 1633),<br>Length of hospital stay
**Notes**	No reply to our emails.

Risk of bias table

Bias	Authors' judgement	Support for judgement
Random sequence generation (selection bias)	Low risk	"... randomized using a computer-generated sequence with a 1:1 allocation and a random block size of 10" (p 1633).
Allocation concealment (selection bias)	Low risk	"Study patients, cardiothoracic surgeons, caregivers, and investigators, including those responsible for data collection, were blinded to the treatment allocation" (p 1633).
Blinding of participants and personnel (performance bias)	Low risk	See above.
Blinding of outcome assessment (detection bias)	Low risk	See above.
Incomplete outcome data (attrition bias)	Low risk	One patient "suffered a ventricular arrhythmia before undergoing cardiothoracic surgery and was excluded" (p 1634).
Selective reporting (reporting bias)	Low risk	
Other bias	Low risk	

Dehghani 2014

Methods

Randomised trial.
http://www.ncbi.nlm.nih.gov/pubmed/24293167
http://dx.doi.org/10.5603/CJ.a2013.0154
http://czasopisma.viamedica.pl/cj/article/view/36075

Participants

Iran, CABG patients, 74 M / 26 F, mean 61 yr (SD 7 yr); 50 vit C / 50 control.

Inclusion: Patients who underwent elective isolated on-pump CABG surgery,
age >50 yr, no history of CABG surgery, taking beta-blocker before and after surgery.

Exclusion: history of any cardiac arrhythmia and/or being under anti-arrhythmic therapy, being under digoxin therapy, having pacemaker, severe CHF and/or LVEF <30%, renal failure, severe hepatic failure, COPD, no occurrence of intra- or post-operative cardiopulmonary arrest, or any degree of cardiac blockade and/or bradycardia.

Interventions

Vit C before the operation:
Dose: 2 g
Method: po
Timing: "All patients took the tablets within 12 hours before surgery" (email 2015-9-9)
Vit C after operation:
Dose: 1 g/d
Method: po
Duration: 5 d.

Placebo: No formal placebo, but the patients received many drugs and it is unlikely that they identified vitamin C among all the other administered drugs. We classify that all the other drugs serve as a functional placebo to vitamin C.

Outcomes

POAF "Postoperative AF was defined as patients who had an episode of AF lasting more than 10 min" [length of the follow-up 5 d] (p 494),
Length of hospital stay

Notes

Additional information was received by emails from Yousef Rezaei on 2015-4-11, 2015-4-22, 2015-9-9, see above and below.

Risk of bias table

Bias	Authors' judgement	Support for judgement
Random sequence generation (selection bias)	Low risk	"All patients were randomized into two groups in a 1:1 ratio using random-number table" (p 493).
Allocation concealment (selection bias)	Low risk	"Neither ward physician nor Holter interpreter were aware of the patients' group. Only one who analyzed data was aware of the patients' group" (email 2015-4-11) and<br><br>"we did not let ward physician and surgeons to know which of patients taking vitamin c or not, except for being informed about the conduction of our trial and prescribing some of patients to take vitamin c. Furthermore, patients were informed that they would be included in our trial to be prescribed vitamin c" (email 2015-4-22).
Blinding of participants and personnel (performance bias)	Low risk	See above.<br>Placebo was not used.
Blinding of outcome assessment (detection bias)	Low risk	See above.
Incomplete outcome data (attrition bias)	Low risk	"There was no patient withdrawal or missing during study. All allocated ones completed study" (email 2015-4-11 and 2015-10-1).
Selective reporting (reporting bias)	Low risk	
Other bias	Low risk	"Our study was funded by Urmia University of Medical Sciences, Iran." (email 2015-10-1)

Donovan 2012

**Methods**	Randomised placebo-controlled 2x2 factorial study with vitamin C and amiodarone<br>https://clinicaltrials.gov/ct2/show/NCT00953212
**Participants**	USA, CABG patients, 230 M / 74 F, mean 65 yr (SD 9 yr); 150 vit C / 154 Placebo<br><br>**Inclusion:** >18 yr, comers for elective or urgent open heart surgery (CABG, Valve repair or replacement, Combined CABG/Valves, CABG/other, Other)<br><br>**Exclusion:** history of AF, emergency surgery, contraindications to study medications, untreated thyroid disease, hepatic failure, pregnancy
**Interventions**	**Vit C before the operation:**<br>**Dose:** 2 g/d<br>**Method:** po<br>**Timing:** 2 g "evening before surgery" and 2 g "morning of surgery"<br>**Vit C after operation:**<br>**Dose:** 2 g/d<br>**Method:** po<br>**Duration:** 5 d<br><br>**Placebo:** was used, but no details
**Outcomes**	POAF<br>Length of hospital stay<br>Length of ICU stay
**Notes**	This trial was identified from ClinicalTrials.gov.<br><br>We were able to contact Dr. Robert S. Kramer who sent the results to us (email 2016-3-23)

Risk of bias table

Bias	Authors' judgement	Support for judgement
Random sequence generation (selection bias)	Low risk	Allocation: Randomized
Allocation concealment (selection bias)	Low risk	Masking: Double Blind (Investigator, Outcomes Assessor)
Blinding of participants and personnel (performance bias)	Low risk	Masking: Double Blind (Investigator, Outcomes Assessor)
Blinding of outcome assessment (detection bias)	Low risk	Masking: Double Blind (Investigator, Outcomes Assessor)
Incomplete outcome data (attrition bias)	Low risk	
Selective reporting (reporting bias)	Low risk	
Other bias	Low risk	

Eslami 2007

**Methods**	Randomised trial.<br>http://www.ncbi.nlm.nih.gov/pubmed/17948074<br>http://www.ncbi.nlm.nih.gov/pmc/articles/PMC1995047
**Participants**	Iran, CABG patients, 67 M / 33 F, mean 60 yr (SD 7 yr), 50 vit C / 50 control.<br><br>**Inclusion:** Isolated CABG patients with age >50 yr and treatment with beta-blockers for a target heart rate of about 60–70 bpm, at least 1 week before surgery.<br><br>**Exclusion:** a history of AF, medication with class I and III antiarrhythmic agents or digoxin, a permanent or temporary pacemaker, any degree of AV block or bradycardia, end stage renal disease, severe pulmonary disease, severe hepatic disease.
**Interventions**	**Vit C before the operation:**<br>**Dose:** 2 g<br>**Method:** po<br>**Timing:** "night before surgery"<br>**Vit C after operation:**<br>**Dose:** 2 g/d<br>**Method:** po<br>**Duration:** 5 d.<br><br>**Placebo:** No formal placebo, but the patients received many medicines and it is unlikely that they identified vitamin C among all the other administered drugs, see below. We classify that all the other drugs serve as a functional placebo to vitamin C.
**Outcomes**	POAF "An episode of atrial fibrillation lasting >10 minutes or the requirement for urgent intervention due to atrial fibrillation" (p 270),<br>Length of hospital stay,<br>Length of ICU stay
**Notes**	Additional information was received by email from Mehdi Mousavi on 2015-4-19, see below.<br><br>"The study was performed as a thesis of cardiology degree and it was supported by Tehran University of medical sciences as a survey project" (email).

Risk of bias table

Bias	Authors' judgement	Support for judgement
Random sequence generation (selection bias)	Low risk	"randomized trial"... "patients were randomly assigned to..." (p 269).<br><br>"The randomization was done with block randomization ... Randomization was done by 1 investigator blinded to the drugs therapy and holter results. It was done with a table of 4 cell block randomization" (email 2015-4-19).
Allocation concealment (selection bias)	Low risk	See above.
Blinding of participants and personnel (performance bias)	Low risk	"The surgeons were blinded. Ascorbic acid prescription and randomization was done by me, blinded to the results of holter and follow up and holter recordings were red by Dr. Eslami who was blinded to everything... A patient who is a candidate for cardiac surgery might take many medications, usually including aspirin, nitrates, statins, possibly ACE inhibitors or ARBs etc., and as the design of our study beta blocker prescription was done to both group, thus 2 groups were receiving lots of drugs and including placebo or not including it in the regimen might not have a serious effect on result of holter monitoring that is an objective observation. Other drugs could work as placebo for control group!" (email 2015-4-19).<br><br>In our authors' judgement, we do not consider that the findings are biased by the lack of formal placebo in the placebo group.
Blinding of outcome assessment (detection bias)	Low risk	"All of the Holter recordings were examined by a single investigator who had been blinded to patients’ group assignments" (p 270).
Incomplete outcome data (attrition bias)	Low risk	"There was no drop out. The study was in-hospital and thus we were able to follow all included patients" (email 2015-4-19).
Selective reporting (reporting bias)	Low risk	

Other bias	Low risk	Funding: “The study was performed as a thesis of cardiology degree and it was supported by Tehran University of medical sciences as a survey project.” (email 2015-4-11)

Healy 2010

**Methods**	Randomised trial.<br>http://dx.doi.org/10.1592/phco.30.10.1086
**Participants**	USA, 30 CABG patients; no data of sex and age distributions.<br><br>**Inclusion:** patients >18yr able to provide informed consent.<br><br>**Exclusion:** persistent or recent AF, or patients who have taken a class I or III antiarrhythmic agent within a predefined period.
**Interventions**	Vitamin C dosage and duration not reported in the abstract<br><br>**Placebo:** No placebo.
**Outcomes**	POAF,<br>Length of hospital stay was measured (N = 60), but not reported.
**Notes**	Published only as an abstract.<br><br>No reply to our emails.

Risk of bias table

Bias	Authors' judgement	Support for judgement
Random sequence generation (selection bias)	Low risk	Title states that the study was "randomized"
Allocation concealment (selection bias)	Unclear risk	?
Blinding of participants and personnel (performance bias)	Unclear risk	?
Blinding of outcome assessment (detection bias)	Unclear risk	?
Incomplete outcome data (attrition bias)	Unclear risk	?
Selective reporting (reporting bias)	High risk	Hospital stay measured but not reported
Other bias	Unclear risk	?

Korantzopoulos 2005

**Methods**	Randomised trial.<br>http://www.ncbi.nlm.nih.gov/pubmed/15982504<br>http://dx.doi.org/10.1016/j.ijcard.2004.12.041
**Participants**	Greece, patients after a successful cardioversion, 26 M / 18 F, mean 68 yr (SD 10 yr); 22 vit C / 22 control. No information about the etiology of AF.<br><br>**Inclusion:** persistent AF (>1 week) scheduled for elective external electrical cardioversion, participants who restored SR for >1 hr were included in the analysis.<br><br>**Exclusion:** thyroid dysfunction, valvular heart disease, left atrial diameter >55 mm, congestive HF (NYHA class >II), recent acute coronary event or revascularization, recent infection, malignancies, WBC dyscrasias, autoimmune or inflammatory diseases, renal failure, or hepatic failure. Patients receiving drugs with antiinflammatory or antioxidant action, apart from statins, as well as multivitamin compounds.
**Interventions**	**Vit C before cardioversion:**<br>**Dose:** 2 g<br>**Method:** po<br>**Timing:** "12 h before cardioversion"<br>**Vit C after operation:**<br>**Dose:** 1 g/d<br>**Method:** po<br>**Duration:** 7 d.<br>Before cardioversion 2 g vit C orally and thereafter 1 g daily for 7 d.<br><br>**Placebo:** No placebo, vitamin C tablets were bought by the patients.
**Outcomes**	Recurrence of AF after a successful cardioversion<br>"Early AF recurrence was defined as relapse into AF within 1 week following successful cardioversion" (p 322).
**Notes**	Additional information was received by email from Panagiotis Korantzopoulos on 2015-4-11, see below.<br><br>"There was no funding. Vitamin C tablets were bought by the patients - very cheap tablets" (email 2015-4-11).

Risk of bias table

Bias	Authors' judgement	Support for judgement
Random sequence generation (selection bias)	Low risk	"randomised in one to one fashion" (p 322).
Allocation concealment (selection bias)	Low risk	"Yes we used allocation concealment" (email 2015-4-11)
Blinding of participants and personnel (performance bias)	Unclear risk	"The physician that was responsible for cardioversion and follow-up of each patient was unaware of the treatment that was assigned to the patient" (p 322). However, "Vitamin C tablets were bougth by the patients - very cheap tablets" (email 2015-4-11).<br><br>In our authors' judgement, we do not consider that the findings are biased by the lack of placebo in the control group.
Blinding of outcome assessment (detection bias)	Low risk	See above and "All measurements were performed blindly to the patients characteristics and treatment" (p 322).
Incomplete outcome data (attrition bias)	Low risk	"In six patients, cardioversion failed to restore sinus rhythm and were excluded from the analysis" (p 322).
Selective reporting (reporting bias)	Low risk	
Other bias	Low risk	Funding: “There was no funding. Vitamin C tablets were bought by the patients - very cheap tablets” (email 2015-4-10)

Papoulidis 2011

Methods

Randomised placebo-controlled trial.

http://www.ncbi.nlm.nih.gov/pubmed/21098510
http://dx.doi.org/10.1510/icvts.2010.240473

Participants

Greece, CABG patients, 120 M / 50 F, mean 73 yr (SD 7 yr); 85 vit C / 85 placebo.

Inclusion: Patients scheduled to undergo elective isolated CABG

Exclusion: <65 yr, preoperative AF, hyperoxaluria, permanent or temporary pacemaker, severe renal or hepatic failure, medication with class I and III antiarrhythmic agents or digoxin, any degree of AV block or bradycardia with a HR <50 bpm, severe pulmonary disease, enlarged left atrium (LA diameter >4.4 cm).

Interventions

Vit C before the operation:
Dose: 2 g
Method: iv
Timing: "3 h prior the initiation of CPB"
Vit C after operation:
Dose: 1 g/d
Method: iv
Duration: 5 d.

Placebo: intravenous administration of 0.9% saline. "Same amount and at the same time" (email).

Outcomes

POAF "The detection of an episode of AF lasting >10 min or the requirement for urgent intervention due to AF" (p 122).
"The outcome was monitored till the day of the discharge, usually day 6-7 postop",
Length of hospital stay,
Length of ICU stay

Notes

Additional information was received by emails from Pavlos Papoulidis on 2015-5-15 and 2015-6-2, see above and below.
"It was more like a self funding" (email).

The authors reported the length of hospital stay as 7.9 (SD 2.2) days in vitamin C group and 9.8 (SD 3.6) days in the placebo group. With the RevMan program, this gives P < 0.0001, whereas the authors published P = 0.04. We adjusted SD = 6.00 days to both groups which leads to P =

0.04 in RevMan.

The authors reported the length of ICU stay as 1.6 (SD 0.9) days in vitamin C group and 2.1 (SD 1.1) days in the placebo group. With the RevMan program, this gives P= 0.0014, whereas the authors published P = 0.05. We adjusted SD = 1.66 days to both groups which leads to P = 0.05 in RevMan.

Risk of bias table

Bias	Authors' judgement	Support for judgement
Random sequence generation (selection bias)	Low risk	"The initial random assignment was by flipping a coin ... to have an equal sample size, we reevaluated our randomization protocol and using a random generator ..." (p 122).
Allocation concealment (selection bias)	Low risk	"During the randomization stage, patients and physicians were not aware of the group to which the participants were allocated" (email 2015-6-2)
Blinding of participants and personnel (performance bias)	Low risk	"During the study, patients and physicians in charge of the treatment, surgeons carrying out the operation, and the physicians interpreting the ECG recordings were all blinded of the study group" (email 2015-6-2)
Blinding of outcome assessment (detection bias)	Low risk	See above
Incomplete outcome data (attrition bias)	Low risk	"No dropouts/withdrawals" (email 2015-5-15)
Selective reporting (reporting bias)	Low risk	
Other bias	Low risk	Funding: “It was more like a self funding.” (email 2015-5-15)

Polymeropoulos 2015

**Methods**	Randomised placebo-controlled trial.<br>https://clinicaltrials.gov/ct2/show/NCT01107730
**Participants**	Greece, CABG patients, 22 CABG patients; 13 M 9 F; Mean age 70 (range 41 to 85); 11 vit C 11 Placebo<br><br>**Inclusion Criteria:** Patients undergoing on-pump cardiac surgery<br><br>**Exclusion Criteria:** off-pump cardiac surgery procedure, AF before the procedure, recent infection and/or infectious endocarditis, neoplasia, chronic renal failure, hepatic failure, autoimmune disease and/or disease that brings about a systematic inflammatory response, thyroid disease, systematic use of supplements that contain vitC or carnitine, use of NSAIDs other than aspirin for a time period up to one month before the procedure.
**Interventions**	**Vit C before the operation:**<br>**Dose:** 2 g/d<br>**Method:** iv<br>**Timing:** "The last dose of vitC was on the evening before" about 12 hours (email 2015-9-9)<br>**Vit C after operation:**<br>**Dose:** 2 g/d<br>**Method:** iv<br>**Duration:** 4 d.<br><br>**Placebo** was saline
**Outcomes**	POAF,<br>Length of hospital stay,<br>Length of ICU stay<br><br>Data for the two latter outcomes were kindly sent to us by Dr. Polymeropoulos as a data set (2015-9-16).
**Notes**	This summary is based on ClinicalTrials.gov document NCT01107730.<br>No full report of the trial was available.<br><br>Additional information was received by email from Evangelos Polymeropoulos on 2015-9-9 and on 2015-9-16, see above and below.

Risk of bias table

Bias	Authors' judgement	Support for judgement
Random sequence generation (selection bias)	Low risk	"Randomization was performed by permutated blocks." (email 2015-9-9)
Allocation concealment (selection bias)	Low risk	Double-blind implies allocation concealment
Blinding of participants and personnel (performance bias)	Low risk	"Double Blind (Subject, Caregiver, Investigator, Outcomes Assessor)"
Blinding of outcome assessment (detection bias)	Low risk	"Double Blind (Subject, Caregiver, Investigator, Outcomes Assessor)"
Incomplete outcome data (attrition bias)	Low risk	"We did not have any dropouts" (email 2015-9-9)
Selective reporting (reporting bias)	Low risk	
Other bias	Low risk	

Rebrova 2012

**Methods**	Randomised trial.<br>http://www.ncbi.nlm.nih.gov/pubmed/22839718<br>http://www.medvestnik.ru/library/article/2904<br>http://www.mv.helsinki.fi/home/hemila/CAF/Rebrova_2012_Rus.pdf<br>**Translation available at:**<br>http://www.mv.helsinki.fi/home/hemila/T12.pdf
**Participants**	Russia, CABG patients, 40 M / 0 F, mean 59 yr (SD 7 yr); 20 vit C / 20 placebo.<br><br>Patients suffering from chronic IHD. All the patients received standard basic treatment before and after operation, including beta-blockers.
**Interventions**	**Vit C before the operation:**<br>**Dose:** 2 g<br>**Method:** po<br>**Timing:** "evening before the operation"<br>**Vit C after operation:**<br>**Dose:** 2 g/d<br>**Method:** po<br>**Duration:** 5 d.<br><br>**No placebo**
**Outcomes**	POAF “AF was defined by sporadic ECG records when the patient feels the palpitations. There was no the recording of the occurrence of AF limited to 5 days, which is the vitamin C administration period.” (email 2015-6-17)<br><br>"After surgery patients were placed in the department of anesthesiology and intensive care. Certainly, in the intensive care department the patients were recorded ECG. As wrote in our article the control of the arrhythmias was performed by ECG monitoring" (email 2015-6-18).
**Notes**	Additional information was received by emails from Tatjana Rebrova on 2015-6-17 and 2015-6-18, see above and below.<br><br>We arranged translation of the text to English.<br>http://www.mv.helsinki.fi/home/hemila/T12.pdf

Risk of bias table

Bias	Authors' judgement	Support for judgement
Random sequence generation (selection bias)	Low risk	"To separate the patients into groups, we used the method of envelope randomization: 20 sealed envelopes contained a piece of paper with the word 'control group', 20 - the inscription 'core group'. The envelopes were mix in a box, and mixed before each withdrawal next envelope. On a certain day we started this research. After the patient signed an informed consent about participation in this research, our researcher took an envelope out of the box and dissected it." (email 2015-6-17)<br><br>"The 'core group' is the group of patients which took vitamin C" (email 2015-6-18)
Allocation concealment (selection bias)	Low risk	See above
Blinding of participants and personnel (performance bias)	Unclear risk	"We did not use placebo.<br><br>Surgeons were informated about to which group patient belonged" (email 2015-6-17)<br><br>"the patients and all physicians knew which groups the patients belonged" (email 2015-6-18)
Blinding of outcome assessment (detection bias)	Unclear risk	See above
Incomplete outcome data (attrition bias)	Low risk	All randomized were included in analysis
Selective reporting (reporting bias)	Low risk	
Other bias	Low risk	

Sadeghpour 2015

**Methods**	Randomised placebo-controlled trial.<br>http://www.ncbi.nlm.nih.gov/pubmed/25789244<br>http://www.ncbi.nlm.nih.gov/pmc/articles/PMC4350190<br><br>Duplicate publication in Farsi (Persian):<br>Moludi J , Keshavarz S , Pakzad R , Sedghi N , Sadeghi T and Alimoradi F.<br>Effect of vitamin C supplementation in the prevention of atrial fibrillation. [Persian]<br>Tehran University Medical Journal, 2016, 73(11), 791<br>http://tumj.tums.ac.ir/browse.php?a_code=A-10-25-5413&sid=1&slc_lang=en
**Participants**	Iran, CABG or valvular surgery patients, 191 M / 99 F, mean 56 yr (SD 14 yr); 113 vit C / 177 placebo.<br><br>**Inclusion:** >18 yr with American Society of Anesthesiologists physical status class II-III and candidacy for CABG or simple congenital valvular disease surgery.<br><br>**Exclusion:** who died within the 1st postoperative day and those who had not received adequate doses of drugs according to our protocol, severe complications (cardiac, respiratory or neurological) or emergent operation.
**Interventions**	**Vit C before the operation:**<br>**Dose:** 2 g<br>**Method:** iv<br>**Timing:** "immediately before surgery"<br>**Vit C after operation:**<br>**Dose:** 1 g/d<br>**Method:** po<br>**Duration:** 4 d.<br><br>**Placebo:** "The patients in the placebo group received an equal number of identical tablets. The placebo tablets and ampoules were prepared in the same shape and size as the original" (p 2). Before surgery: "The Vit C was given in the operating room along with the other infusions by anesthesiologist technician" (email 2015-5-11).
**Outcomes**	POAF "AF rhythm was defined by 10 min period of AF rhythm in the ECG monitoring for the first 3-4th days after the cardiac surgery or when it was detected in 12- lead ECG in day 4 or 5 after the surgery (Continuous ECG monitoring for day 3-4 and daily ECG on day 4 and 5 based on our protocol) or whenever symptoms occurred and was documented by ECG monitoring" and the

	recording was limited to 5 days (email 2015-5-12),<br><br>Length of hospital stay,<br>Length of ICU stay
**Notes**	Additional information was received by email from Anita Sadeghpour on 2015-5-12 and 2015-10-1, see below and above.<br><br>The authors reported the length of hospital stay as 10.17 (SD 4.63) days in vitamin C group and 12 (SD 4.51) days in the placebo group. With the RevMan program, this gives $P < 0.0009$, whereas the authors published $P = 0.01$. We adjusted SD = 5.90 days to both groups which leads to $P = 0.01$ in RevMan.

Risk of bias table

Bias	Authors' judgement	Support for judgement
Random sequence generation (selection bias)	Unclear risk	"The study population was randomized one day before surgery to two groups (By using www.randomizer.org) ... The method of randomization was balanced block with an allocation sequence based on a block size of eight" (p 2).<br><br>However, the sizes of the groups 113 vit C / 177 placebo are not consistent with block randomization. Therefore we exclude this study in our sensitivity analysis.
Allocation concealment (selection bias)	Low risk	"Both the patients and the hospital staff were blind to the treatment allocation" (p 2).
Blinding of participants and personnel (performance bias)	Low risk	See above.<br><br>"The Vit C was given in the operating room along with the other infusions by anesthesiologist technician" (email 2015-5-12).
Blinding of outcome assessment (detection bias)	Low risk	See above.
Incomplete outcome data (attrition bias)	Low risk	
Selective reporting (reporting bias)	Low risk	
Other bias	Low risk	"We paid it by ourselves besides getting help from the Rajaei cardiovascular research center." (email 2015-10-1)

Samadikhah 2014

**Methods**	Randomised placebo-controlled trial.<br><br>http://www.ncbi.nlm.nih.gov/pubmed/24409416<br>http://www.ncbi.nlm.nih.gov/pmc/articles/PMC3885376
**Participants**	Iran, CABG patients; 82 M / 38 F, mean 61 yr (SD 11 yr); 60 vit C / 60 placebo.<br><br>**Inclusion:** patients who were scheduled to undergo CABG surgery.<br><br>**Exclusion:** AF, left atrial hypertrophy, heart valve disease, myocardial infarction and ejection fraction of left ventricle <40%.
**Interventions**	**Vit C before the operation:**<br>**Dose:** 2 g<br>**Method:** po<br>**Timing:** "in operation day"<br>**Vit C after operation:**<br>**Dose:** 1 g/d<br>**Method:** po<br>**Duration:** 5 d.<br><br>**Placebo:** "placebo with the same dose were used for controls" (p 98). "The tablets were prepared by the pharmacy faculty with the size, weight and shape to that of the vit C tablets we used in the study" (email 2015-4-19).
**Outcomes**	POAF "AF diagnosis was based on EKG findings (consisting a standard 12 lead EKG with a long lead II)" based on 5-day followup (email 2015-4-19),<br><br>Length of hospital stay and length of ICU stay were measured and mentioned in the report, but data were not reported, and the data were not available for us, when we contacted Dr. Golzari.
**Notes**	Additional information was received by email from Samad Golzari on 2015-4-19, see below.

Risk of bias table

Bias	Authors' judgement	Support for judgement
Random sequence generation (selection bias)	Low risk	"for a random double-blind clinical trial" (p 98); "Randlist software was used" (email 2015-4-19).
Allocation concealment (selection bias)	Low risk	"double blind" (p 98) implies allocation concealment and "yes" (email 2015-4-19) to our question describing the concept of allocation concealment, and whether it was used.
Blinding of participants and personnel (performance bias)	Low risk	"double blind" (p 98) and "Patients did not know if the medication they received was vit C or placebo. Neither was the physician giving the medications to the patients aware of the content of the tablets" (email 2015-4-19), and surgeons carrying out the CABG operation, physicians in the ICU, physicians assessing the ECG: "All above mentioned people were blinded as the medication was given by a single person who was also blinded to the medications" (email 2015-4-19).
Blinding of outcome assessment (detection bias)	Low risk	See above.
Incomplete outcome data (attrition bias)	Low risk	"there were no dropouts" (email 2015-4-19).
Selective reporting (reporting bias)	High risk	ICU stay and Hospital stay were measured but not reported since they were not significant.
Other bias	Low risk	"All our studies are funded by our university: Tabriz University of Medical Sciences, Tabriz, Iran" (email 2015-10-1)

Sarzaeem 2014

**Methods**	Randomised placebo-controlled trial.<br><br>http://tumj.tums.ac.ir/browse.php?a_id=5853&sid=1&slc_lang=en<br>http://tumj.tums.ac.ir/browse.php?a_id=5853&slc_lang=en&sid=1&ftxt=1<br>**Translation at:**<br>http://www.mv.helsinki.fi/home/hemila/T14.pdf
**Participants**	Iran, CABG patients, 118 M / 52 F, mean 59 yr (SD 10 yr) ; 85 vit C / 85 placebo.<br><br>**Inclusion:** Patients with coronary artery disease (in angiography) who were candidates for coronary artery bypass<br><br>**Exclusion:** >80 yr, AF before surgery; valvular heart disease, arrhythmia, or cardiac conduction block of any degree; pacemaker, chronic lung, liver, or kidney disease; other heart surgeries along with CABG, history of antiarrhythmic drug consumption, sick sinus syndrome; symptoms or history of urinary calculi, vitamin C consumption during the last 3 months.
**Interventions**	**Vit C before the operation:**<br>**Dose:** 2 g<br>**Method:** iv<br>**Timing:** "12 h before the procedure"<br>**Vit C after operation:**<br>**Dose:** 1 g/d<br>**Method:** iv<br>**Duration:** 5 d.<br><br>**Placebo:** "patients in the control group received placebo (normal saline intravenously)" (abstract).
**Outcomes**	POAF,<br>Length of hospital stay,<br>Length of ICU stay

**Notes**	The report was published in Farsi (Persian) with an abstract in English.<br><br>No reply to our emails.<br><br>We arranged translation of the report to English: http://www.mv.helsinki.fi/home/hemila/T14.pdf<br><br>In table, the results were reported to one digit. The text section reports that there was a 1.53 day difference between groups in the length of hospital stay, and a 0.49 day difference in the length of ICU stay was Therefore, to make the comparison more accurate, we adjusted the vitamin C hospital stay to 6.67 d and ICU stay to 2.51 d, keeping the placebo group values as in the table.

Risk of bias table

Bias	Authors' judgement	Support for judgement
Random sequence generation (selection bias)	Low risk	"using a table of random numbers are divided into intervention and control groups to receive placebo or vitamin C" (abstract).
Allocation concealment (selection bias)	Low risk	Double-blind implies allocation concealment
Blinding of participants and personnel (performance bias)	Low risk	"this double-blind, parallel clinical trial" (Abstract) … "The present study was a double-blind parallel group clinical trial, because neither the patients nor the health care workers were aware of the medications in the infusions" (Methods)-
Blinding of outcome assessment (detection bias)	Low risk	See above.
Incomplete outcome data (attrition bias)	Low risk	
Selective reporting (reporting bias)	Low risk	
Other bias	Low risk	

van Wagoner 2003

**Methods**	Randomised placebo-controlled trial.<br><br>2003 Abstract (ref 27):<br>http://www.mv.helsinki.fi/home/hemila/CAF/vanWagoner2003.pdf<br><br>Brief summary also in a 2008 paper (ref 6):<br>http://www.ncbi.nlm.nih.gov/pubmed/18791466<br>http://dx.doi.org/10.1097/FJC.0b013e31817f9398
**Participants**	USA, CABG patients 400 randomized, 338 analyzed; age 63 yr; 177 vit C / 169 placebo<br><br>**Inclusion:** Patients undergoing CABG.<br><br>**Exclusion:** Patients with a history of AF, combined surgery (valve+CABG).
**Interventions**	**Vit C before the operation:**<br>**Dose:** 2 g<br>**Method:** po<br>**Timing:** "night before surgery"<br>**Vit C after operation:**<br>**Dose:** 1 g/d<br>**Method:** po<br>**Duration:** 5 d.<br>"the same dosing strategy" as in Carnes 2001<br><br>**Placebo**
**Outcomes**	POAF,<br>length of hospital stay
**Notes**	Reported very briefly within a review in Van Wagoner 2008.<br>Additional information was received by email from David van Wagoner on 2015-9-2 and 2015-9-25, see below.<br>No full report was available.<br>We contacted Dr. van Wagoner, and he sent the results to us (2015-9-28).<br><br>A summary of the results had been reported in 2013 in an abstract to Heart Rhytm Society

Risk of bias table

Bias	Authors' judgement	Support for judgement
Random sequence generation (selection bias)	Low risk	"randomized, blinded trial" (p 308) "there was a randomization table that had been created" (email 2015-9-25). Randomization was done in pairs with SAS procedure PLAN (email 2015-9-29)
Allocation concealment (selection bias)	Low risk	"double blind" implies allocation concealment
Blinding of participants and personnel (performance bias)	Low risk	"double-blind ... Our investigational pharmacy did the blinding, so all patients and staff were blinded to the treatment." (email 2015-9-2)
Blinding of outcome assessment (detection bias)	Low risk	"double-blind ... Our investigational pharmacy did the blinding, so all patients and staff were blinded to the treatment." (email 2015-9-2)
Incomplete outcome data (attrition bias)	Low risk	"Of the 400 total patients enrolled, 54 were dropped from analysis as they either did not go to surgery, surgery was delayed, they had a combined procedure (valve + CABG), or it was found that they had a history of AF after enrollment." (email 2015-9-28)
Selective reporting (reporting bias)	Low risk	
Other bias	Low risk	

Reasons for excluding 8 studies

Albiez 2003

**Reason for exclusion**	A trial on vitamin C for cardiac surgery patients, but no data on POAF.

Basili 2010

**Reason for exclusion**	A trial on vitamin C for cardiac surgery patients, but no data on POAF.

Carnes 2001

**Reason for exclusion**	Vitamin C administered to a group of cardiac surgery patients. Data about POAF, but comparison to historical controls.

Dingchao 1994

**Reason for exclusion**	No description whether the trial was randomised.<br>A trial on vitamin C for cardiac surgery patients, but no data on POAF.

Ebade 2014

**Reason for exclusion**	Method of allocation not described. Not clear that the study was a randomized trial. We did not get responses to several emails to two authors. The study reported the incidence of POAF in vitamin C and control groups.

Ibrahim 2010

**Reason for exclusion**	Not a parallel comparison study. 142 patients were administered vitamin C before surgery and the incidence of POAF was compared with 1589 historical controls.

Oktar 2001

**Reason for exclusion**	A trial on vitamin C for cardiac surgery patients, but no data on POAF.

Wang 2014

**Reason for exclusion**	A trial on vitamin C for cardiac surgery patients, but no data on POAF.

References for excluded studies

Albiez 2003

* Albiez GA, Preiss DU, Cap, M, Tollenaere PL, Wartenberg-Demand A. High-dose intravenous ascorbic acid reduces platelet loss and ischemic events during coronary bypass surgery - a single-centre, double-blind, randomised study. In: Perfusion. Vol. 16. 2003:4.

Albiez GA, Preiss DU, Jaehnchen E,Cap M, Tollenaere PJ,. High Doses of Ascorbic Acid Diminish Platelet Loss and Accelerate Return to Normal Following Cardiopulmonary Bypass [Abstract]. In: American Society for Anesthesiologists 2000 Annual Meeting Abstracts. 2000:A-177.

Basili 2010

Basili S, Tanzilli G, Mangieri E, Raparelli V, Di Santo S, Pignatelli P, et al.. Intravenous ascorbic acid infusion improves myocardial perfusion grade during elective percutaneous coronary intervention: relationship with oxidative stress markers. Journal of the American College of Cardiology cardiovascular interventions 2010;3(2):221-9. [DOI: 10.1016/j.jcin.2009.10.025; PubMed: 20170881]
http://dx.doi.org/10.1016/j.jcin.2009.10.025

Carnes 2001

Carnes CA, Chung MK, Nakayama T, Nakayama H, Baliga RS, Piao S, et al. Ascorbate attenuates atrial pacing-induced peroxynitrite formation and electrical remodeling and decreases the incidence of postoperative atrial fibrillation. Circulatory Research 2001;89(6):E32-8. [DOI: 10.1161/hh1801.097644; PubMed: 11557745]
http://www.ncbi.nlm.nih.gov/pubmed/11557745
http://dx.doi.org/10.1161/hh1801.097644

Dingchao 1994

Dingchao H, Zhiduan Q, Liye H, Xiaodong F. The protective effects of high-dose ascorbic acid on myocardium against reperfusion injury during and after cardiopulmonary bypass. Thoracic and cardiovascular surgeon 1994;42(5):276-8. [DOI: 10.1055/s-2007-1016504; PubMed: 7863489]
http://www.ncbi.nlm.nih.gov/pubmed/7863489
http://dx.doi.org/10.1055/s-2007-1016504

Ebade 2014

Ebade A, Tahaa WS, Saleh RH, Fawzy A. Ascorbic acid versus magnesium for the prevention of atrial fibrillation after coronary artery bypass grafting surgery. Egyptian Journal of Cardiothoracic Anesthesia 2014;8:59-65. [Other: http://www.ejca.eg.net/article.asp?issn=1687-9090;year=2014;volume=8;issue=2;spage=59;epage=65;aulast=Ebade]
http://dx.doi.org/10.4103/1687-9090.143259

Ibrahim 2010

Ibrahim TM, Hill PC, Bafi AS, Kanda LT, Ellis JL, Lowery RC, et al. The effect of vitamin C on the incidence of atrial fibrillation after major cardiac surgery [Abstract]. Chest 2010;138(4):503A. [DOI: 10.1378/chest.10443]
http://dx.doi.org/10.1378/chest.10443
http://journal.publications.chestnet.org/article.aspx?articleID=1087240

Oktar 2001

Oktar GL, Sinci V, Kalaycioglu S, Soncul H, Gökgöz L, Halit V, Ersöz A. Biochemical and hemodynamic effects of ascorbic acid and alpha-tocopherol in coronary artery surgery. Scandinavian Journal of Clinical and Laboratory Investigation. 2001;61(8):621-9. [DOI: 10.1080/003655101753267982; PubMed: 11768322]
http://www.ncbi.nlm.nih.gov/pubmed/11768322
http://dx.doi.org/10.1080/003655101753267982

Wang 2014

Wang ZJ, Hu WK, Liu YY, Shi DM, Cheng WJ, Guo YH, et al. The effect of intravenous vitamin C infusion on periprocedural myocardial injury for patients undergoing elective percutaneous coronary intervention. Canadian Journal of Cardiology 2014;30(1):96-101. [DOI: 10.1016/j.cjca.2013.08.018; PubMed: 24365194]
http://www.ncbi.nlm.nih.gov/pubmed/24365194
http://dx.doi.org/10.1016/j.cjca.2013.08.018
