# Supplementary material for: Vitamin C for preventing atrial fibrillation in high risk patients: a systematic review and meta-analysis
Source: BMC Cardiovasc Disord. 2017 Feb 1;17:49. doi: 10.1186/s12872-017-0478-5 (PMC5286679; doi:10.1186/s12872-017-0478-5)

Vitamin C for preventing atrial fibrillation in high risk patients: a systematic review and meta-analysis

Harri Hemilä and Timo Suonsyrjä

BMC Cardiovascular Disorders
https://bmccardiovascdisord.biomedcentral.com/

DOI 10.1186/s12872-017-0478-5
https://dx.doi.org/10.1186/s12872-017-0478-5

Additional file 2

version 2017-1-27

Harri Hemilä
Department of Public Health, POB 20
University of Helsinki
Tukholmankatu 8 B 2B
FI-00014 Helsinki
Finland

E-mail: harri.hemila@helsinki.fi
Home page: http://www.mv.helsinki.fi/home/hemila

Contents

Page

2 Transformation of hospital stay to %-scale

3 Transformation of ICU data from days to %-scale

4 AF in the 10 non-US trials

5 AF in the 10 non-US trials: exclusion of 4 trials with some concerns about randomization and blinding

6 Comparison of the 5 US and 5 Iran POAF trials

7 Hospital stay in the non-US trials

8 Hospital stay in the non-US trials: exclusion of 1 trial with some concerns about blinding

Transformation of hospital stay to %-scale

Reported hospital stay in days

Hospital stay	Days					
	Vit C			Placebo		
	mean	SD	N	mean	SD	N
Antonic	9.37	5.27	52	8.77	5.27	53
Bjordahl	10.4	4.0	89	11.7	7.1	96
Colby	14.6	17.6	13	10.2	4.9	11
Dehghani	5.32	0.59	50	5.74	1.3	50
Donovan	6.8	3.6	150	6.8	4.9	154
Eslami	6.54	3.24	50	7.08	3.45	50
Papoulidis	7.9	6.0	85	9.8	6.0	85
Polymeropoulos	11.27	5.31	11	12.73	6.6	11
Sadeghpour	10.17	5.9	113	12.0	5.9	177
Sarzaeem	6.67	1.5	85	8.2	2.3	85
vanWagoner	6.53	3.15	172	6.62	3.15	166

Calculated hospital stay with Placebo group mean = 100%
These values are used for the primary analysis

	Vit C			**Placebo**		
	mean %	SD %	N	**mean %**	SD %	N
Antonic	106.84	60	52	**100**	60	53
Bjordahl	88.89	34	89	**100**	61	96
Colby	143.14	173	13	**100**	48	11
Dehghani	92.68	10	50	**100**	23	50
Donovan	100.0	53	150	**100**	72	154
Eslami	92.37	46	50	**100**	49	50
Papoulidis	80.61	61	85	**100**	61	85
Polymeropoulos	88.53	42	11	**100**	52	11
Sadeghpour	84.75	49	113	**100**	49	177
Sarzaeem	81.34	18	85	**100**	28	85
vanWagoner	98.63	48	172	**100**	48	166

For example,
In the Antonic (2016) trial,
vitamin C group duration is 9.37/8.77 = 1.0684 =106.84% of the placebo group duration

Transformation of ICU data from days to %-scale

Reported ICU stay

ICU stay (days)	Vit C mean	SD	N	Placebo mean	SD	N
Antonic	1.5	1.3	52	1.3	1.3	53
Bjordahl	3.7	2.2	89	4.3	2.9	96
Colby	4.43	10.41	13	2.04	1.8	11
Dehghani	1.79	0.31	50	2.1	0.61	50
Donovan	2,0	1.3	150	1.9	1.5	154
Eslami	2.3	1.6	50	2.6	1.48	50
Papoulidis	1.6	1.66	85	2.1	1.66	85
Sadeghpour	3.42	1.06	113	3.43	1.09	177
Sarzaeem	2.51	1.4	85	3,0	1.6	85

Calculated stay with Placebo group mean = 100%
These values are used for the analysis

	Vit C mean %	SD %	N	**Placebo mean %**	SD %	N
Antonic	115.38	100	52	**100**	100	53
Bjordahl	86.05	51	89	**100**	67	96
Colby	217.59	511	13	**100**	88	11
Dehghani	85.24	15	50	**100**	29	50
Donovan	105.26	68	150	**100**	79	154
Eslami	88.46	62	50	**100**	57	50
Papoulidis	76.19	79	85	**100**	79	85
Sadeghpour	99.71	31	113	**100**	32	177
Sarzaeem	83.67	47	85	**100**	53	85

AF in the 10 non-US trials

If the 5 US trials are removed, the remaining 10 non-US trials are not heterogeneous, I^2 = 38% (P = 0.10), and the pooled estimate indicates a **45% decrease (95% CI 35% to 54%; Z = 6.60, P = 10^{-10})** in the occurrence of AF.

	Vitamin C		Control			Risk Ratio
Study or Subgroup	Events	Total	Events	Total	Weight	M-H, Fixed, 95% CI
**1.1.1 POAF trials in the USA**						
Bjordahl 2012	27	89	29	96	0.0%	1.00 [0.65, 1.56]
Colby 2011	4	13	5	11	0.0%	0.68 [0.24, 1.92]
Donovan 2012	58	150	48	154	0.0%	1.24 [0.91, 1.69]
Healy 2010	5	19	7	11	0.0%	0.41 [0.17, 0.99]
van Wagoner 2003	44	177	41	169	0.0%	1.02 [0.71, 1.48]
**Subtotal (95% CI)**		**0**		**0**		**Not estimable**
Total events	0		0			
Heterogeneity: Not applicable						
Test for overall effect: Not applicable						
**1.1.2 POAF trials Greece, Russia, Slovenia**						
Antonic 2016	7	52	10	53	4.4%	0.71 [0.29, 1.73]
Papoulidis 2011	38	85	52	85	23.1%	0.73 [0.55, 0.98]
Polymeropoulos 2015	4	11	5	11	2.2%	0.80 [0.29, 2.21]
Rebrova 2012	1	20	4	20	1.8%	0.25 [0.03, 2.05]
**Subtotal (95% CI)**		**168**		**169**	**31.5%**	**0.71 [0.54, 0.93]**
Total events	50		71			
Heterogeneity: Chi² = 1.05, df = 3 (P = 0.79); I² = 0%						
Test for overall effect: Z = 2.52 (P = 0.01)						
**1.1.3 POAF trials in Iran**						
Dehghani 2014	4	50	16	50	7.1%	0.25 [0.09, 0.70]
Eslami 2007	2	50	13	50	5.8%	0.15 [0.04, 0.65]
Sadeghpour 2015	40	113	99	177	34.3%	0.63 [0.48, 0.84]
Samadikhah 2014	6	60	15	60	6.7%	0.40 [0.17, 0.96]
Sarzaeem 2014	11	85	25	85	11.1%	0.44 [0.23, 0.84]
**Subtotal (95% CI)**		**358**		**422**	**64.9%**	**0.49 [0.39, 0.62]**
Total events	63		168			
Heterogeneity: Chi² = 7.62, df = 4 (P = 0.11); I² = 48%						
Test for overall effect: Z = 5.82 (P < 0.00001)						
**1.1.4 AF recurrence after a successful cardioversion**						
Korantzopoulos 2005	1	22	8	22	3.6%	0.13 [0.02, 0.92]
**Subtotal (95% CI)**		**22**		**22**	**3.6%**	**0.13 [0.02, 0.92]**
Total events	1		8			
Heterogeneity: Not applicable						
Test for overall effect: Z = 2.04 (P = 0.04)						
**Total (95% CI)**		**548**		**613**	**100.0%**	**0.55 [0.46, 0.65]**
Total events	114		247			
Heterogeneity: Chi² = 14.59, df = 9 (P = 0.10); I² = 38%						
Test for overall effect: Z = 6.60 (P < 0.00001)						
Test for subgroup differences: Chi² = 6.10, df = 2 (P = 0.05), I² = 67.2%						

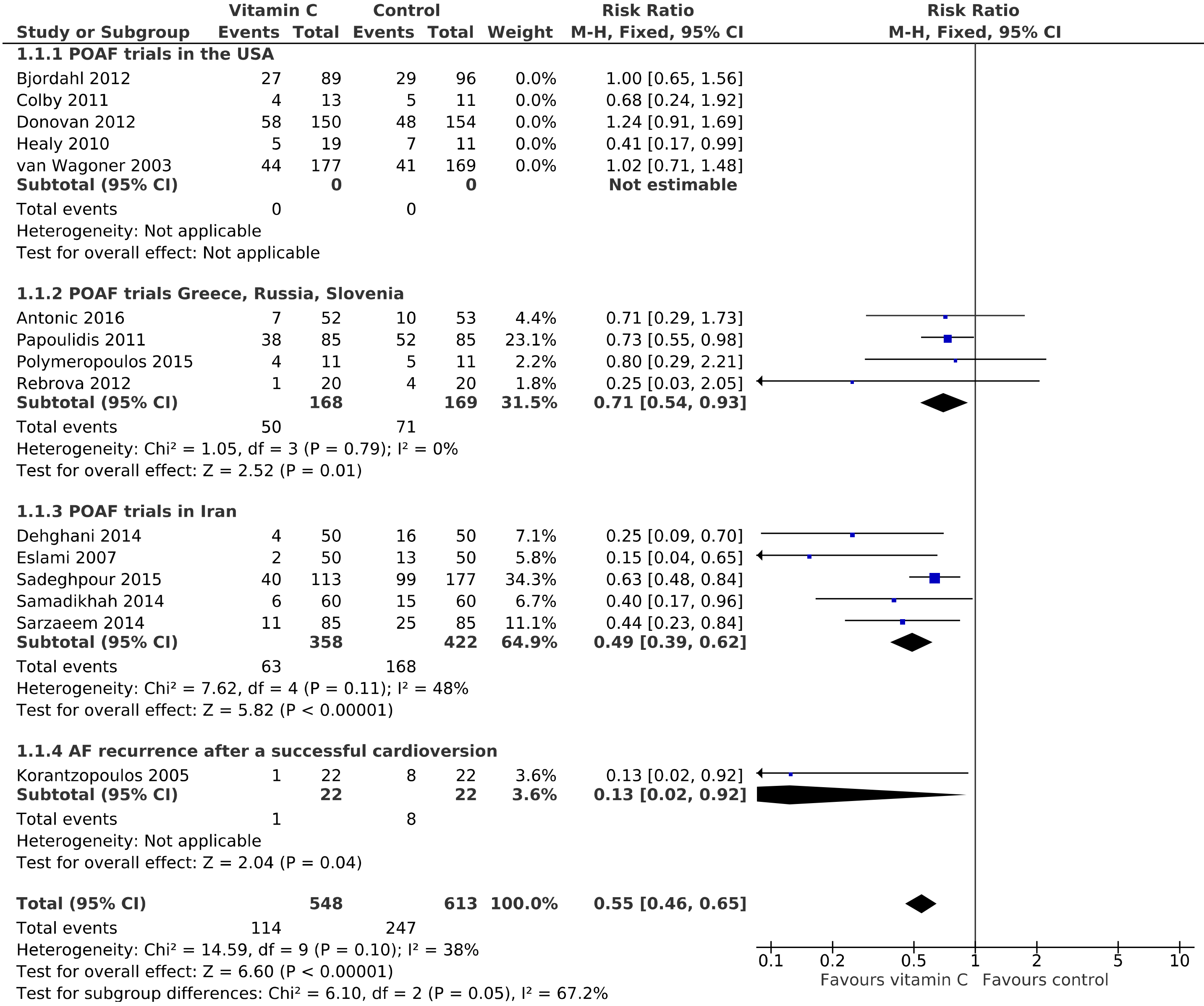

Exclusion of 4 trials with some concerns about randomization and blinding

If we further remove 4 non-US trials that had some concerns about randomization or blinding [23,24,31,32], the effect estimate is essentially the same leading to **48% decrease (95% CI 34% to 60%; Z = 5.30, $P = 10^{-7}$)** in the incidence of AF.

Study or Subgroup	Vitamin C Events	Vitamin C Total	Control Events	Control Total	Weight	Risk Ratio M-H, Fixed, 95% CI
**1.1.1 POAF trials in the USA**						
Bjordahl 2012	27	89	29	96	0.0%	1.00 [0.65, 1.56]
Colby 2011	4	13	5	11	0.0%	0.68 [0.24, 1.92]
Donovan 2012	58	150	48	154	0.0%	1.24 [0.91, 1.69]
Healy 2010	5	19	7	11	0.0%	0.41 [0.17, 0.99]
van Wagoner 2003	44	177	41	169	0.0%	1.02 [0.71, 1.48]
**Subtotal (95% CI)**		**0**		**0**		**Not estimable**
Total events	0		0			
Heterogeneity: Not applicable						
Test for overall effect: Not applicable						
**1.1.2 POAF trials Greece, Russia, Slovenia**						
Antonic 2016	7	52	10	53	0.0%	0.71 [0.29, 1.73]
Papoulidis 2011	38	85	52	85	41.3%	0.73 [0.55, 0.98]
Polymeropoulos 2015	4	11	5	11	4.0%	0.80 [0.29, 2.21]
Rebrova 2012	1	20	4	20	0.0%	0.25 [0.03, 2.05]
**Subtotal (95% CI)**		**96**		**96**	**45.2%**	**0.74 [0.56, 0.97]**
Total events	42		57			
Heterogeneity: Chi² = 0.03, df = 1 (P = 0.87); I² = 0%						
Test for overall effect: Z = 2.14 (P = 0.03)						
**1.1.3 POAF trials in Iran**						
Dehghani 2014	4	50	16	50	12.7%	0.25 [0.09, 0.70]
Eslami 2007	2	50	13	50	10.3%	0.15 [0.04, 0.65]
Sadeghpour 2015	40	113	99	177	0.0%	0.63 [0.48, 0.84]
Samadikhah 2014	6	60	15	60	11.9%	0.40 [0.17, 0.96]
Sarzaeem 2014	11	85	25	85	19.8%	0.44 [0.23, 0.84]
**Subtotal (95% CI)**		**245**		**245**	**54.8%**	**0.33 [0.22, 0.52]**
Total events	23		69			
Heterogeneity: Chi² = 2.30, df = 3 (P = 0.51); I² = 0%						
Test for overall effect: Z = 4.93 (P < 0.00001)						
**1.1.4 AF recurrence after a successful cardioversion**						
Korantzopoulos 2005	1	22	8	22	0.0%	0.13 [0.02, 0.92]
**Subtotal (95% CI)**		**0**		**0**		**Not estimable**
Total events	0		0			
Heterogeneity: Not applicable						
Test for overall effect: Not applicable						
**Total (95% CI)**		**341**		**341**	**100.0%**	**0.52 [0.40, 0.66]**
Total events	65		126			
Heterogeneity: Chi² = 11.44, df = 5 (P = 0.04); I² = 56%						
Test for overall effect: Z = 5.30 (P < 0.00001)						
Test for subgroup differences: Chi² = 8.97, df = 1 (P = 0.003), I² = 88.9%						

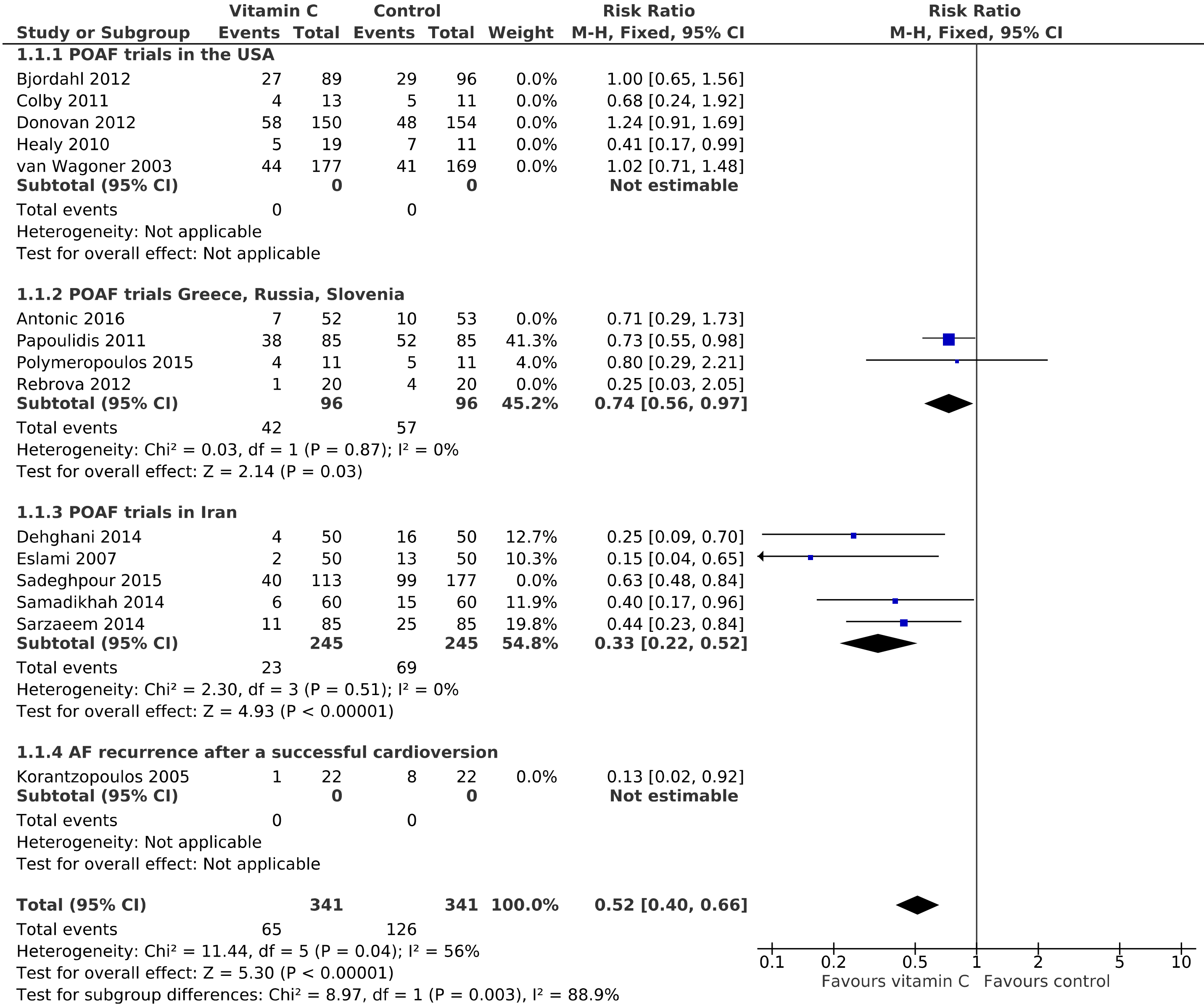

Comparison of the 5 US and 5 Iran POAF trials

In a direct comparison of the 5 US POAF trials against the 5 Iran POAF trials, there is very strong evidence of heterogeneity with **I^2 = 95%; P = 10^{-5} in heterogeneity test**.

	Vitamin C		Control			Risk Ratio
**Study or Subgroup**	**Events**	**Total**	**Events**	**Total**	**Weight**	**M-H, Fixed, 95% CI**
**1.3.1 POAF trials in the USA**						
Bjordahl 2012	27	89	29	96	10.0%	1.00 [0.65, 1.56]
Colby 2011	4	13	5	11	2.0%	0.68 [0.24, 1.92]
Donovan 2012	58	150	48	154	17.1%	1.24 [0.91, 1.69]
Healy 2010	5	19	7	11	3.2%	0.41 [0.17, 0.99]
van Wagoner 2003	44	177	41	169	15.1%	1.02 [0.71, 1.48]
**Subtotal (95% CI)**		**448**		**441**	**47.4%**	**1.04 [0.86, 1.27]**
Total events	138		130			
Heterogeneity: Chi² = 6.20, df = 4 (P = 0.18); I² = 35%						
Test for overall effect: Z = 0.41 (P = 0.68)						
**1.3.3 POAF trials in Iran**						
Dehghani 2014	4	50	16	50	5.8%	0.25 [0.09, 0.70]
Eslami 2007	2	50	13	50	4.7%	0.15 [0.04, 0.65]
Sadeghpour 2015	40	113	99	177	27.8%	0.63 [0.48, 0.84]
Samadikhah 2014	6	60	15	60	5.4%	0.40 [0.17, 0.96]
Sarzaeem 2014	11	85	25	85	9.0%	0.44 [0.23, 0.84]
**Subtotal (95% CI)**		**358**		**422**	**52.6%**	**0.49 [0.39, 0.62]**
Total events	63		168			
Heterogeneity: Chi² = 7.62, df = 4 (P = 0.11); I² = 48%						
Test for overall effect: Z = 5.82 (P < 0.00001)						
**Total (95% CI)**		**806**		**863**	**100.0%**	**0.75 [0.65, 0.87]**
Total events	201		298			
Heterogeneity: Chi² = 31.50, df = 9 (P = 0.0002); I² = 71%						
Test for overall effect: Z = 3.71 (P = 0.0002)						
Test for subgroup differences: Chi² = 22.49, df = 1 (P < 0.00001), I² = 95.6%						

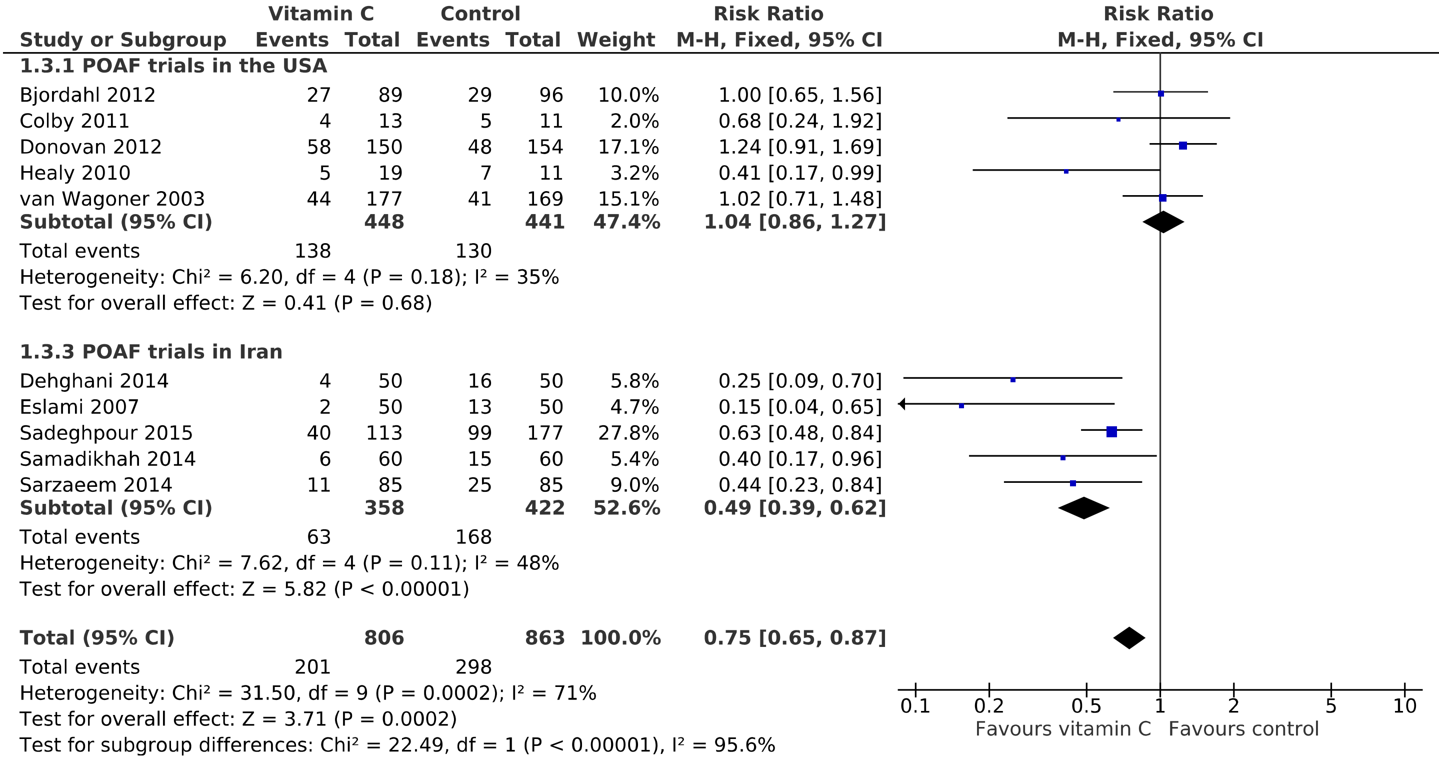

Hospital stay in the non-US trials

In the non-US cardiac surgery trials, vitamin C decreased the length of hospital stay by **12.6% (95% CI 8.4% to 16.8%; Z = 5.87, P = 10^{-8}**).

	Vitamin C			Control				Mean Difference
**Study or Subgroup**	**Mean**	**SD**	**Total**	**Mean**	**SD**	**Total**	**Weight**	**IV, Fixed, 95% CI**
**2.1.1 US trials, oral vitamin C in all**								
Bjordahl 2012	89	34	89	100	61	96	0.0%	-11.00 [-25.10, 3.10]
van Wagoner 2003	99	48	172	100	48	166	0.0%	-1.00 [-11.24, 9.24]
Donovan 2012	100	53	150	100	72	154	0.0%	0.00 [-14.19, 14.19]
Colby 2011	143	172	13	100	48	11	0.0%	43.00 [-54.71, 140.71]
**Subtotal (95% CI)**			**0**			**0**		**Not estimable**
Heterogeneity: Not applicable								
Test for overall effect: Not applicable								
**2.1.2 Oral vitamin C in non-US trials**								
Eslami 2007	92.37	46	50	100	49	50	5.1%	-7.63 [-26.26, 11.00]
Dehghani 2014	92.68	10	50	100	23	50	36.6%	-7.32 [-14.27, -0.37]
**Subtotal (95% CI)**			**100**			**100**	**41.7%**	**-7.36 [-13.87, -0.84]**
Heterogeneity: Chi² = 0.00, df = 1 (P = 0.98); I² = 0%								
Test for overall effect: Z = 2.21 (P = 0.03)								
**2.1.3 Intravenous vitamin C in non-US trials**								
Papoulidis 2011	80.61	61	85	100	61	85	5.3%	-19.39 [-37.73, -1.05]
Sarzaeem 2014	81.34	18	85	100	28	85	35.3%	-18.66 [-25.74, -11.58]
Sadeghpour 2015	84.75	49	113	100	49	177	13.2%	-15.25 [-26.81, -3.69]
Polymeropoulos 2015	88.53	42	11	100	52	11	1.1%	-11.47 [-50.97, 28.03]
Antonic 2016	106.8	60	52	100	60	53	3.4%	6.80 [-16.15, 29.75]
**Subtotal (95% CI)**			**346**			**411**	**58.3%**	**-16.35 [-21.85, -10.84]**
Heterogeneity: Chi² = 4.52, df = 4 (P = 0.34); I² = 11%								
Test for overall effect: Z = 5.82 (P < 0.00001)								
**Total (95% CI)**			**446**			**511**	**100.0%**	**-12.60 [-16.80, -8.39]**
Heterogeneity: Chi² = 8.78, df = 6 (P = 0.19); I² = 32%								
Test for overall effect: Z = 5.87 (P < 0.00001)								
Test for subgroup differences: Chi² = 4.27, df = 1 (P = 0.04), I² = 76.6%								

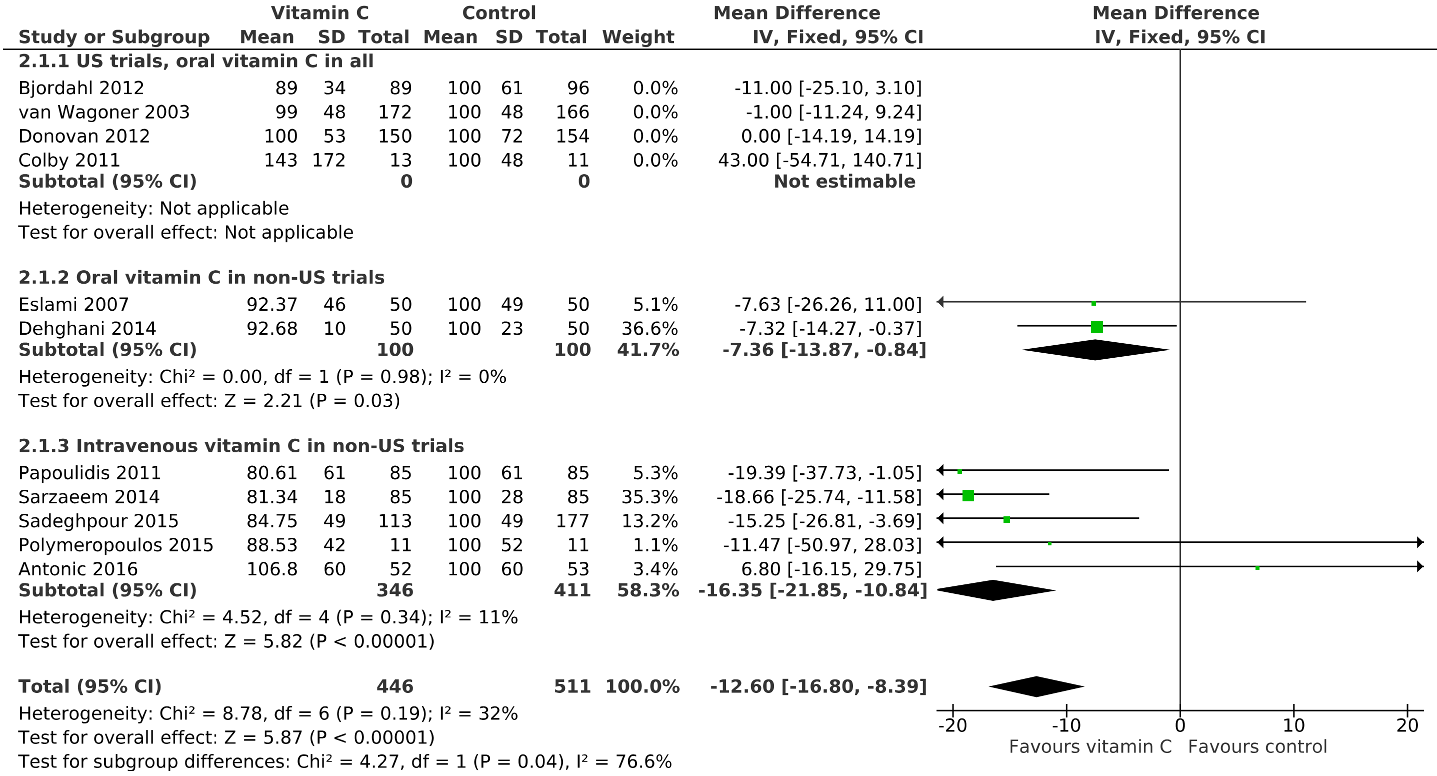

Exclusion of 1 trial with some concerns about blinding

Only 1 of the non-US trials had some concerns about blinding [23], and its exclusion had only a small effect on the pooled effect estimate leading to **13.3% decrease (12.1% to 23.4%;**
Z = 6.08, P = 10^{-9}) decrease in the length of hospital stay.

Study or Subgroup	Vitamin C Mean	Vitamin C SD	Vitamin C Total	Control Mean	Control SD	Control Total	Weight	Mean Difference IV, Fixed, 95% CI
**2.1.1 US trials, oral vitamin C in all**								
Bjordahl 2012	89	34	89	100	61	96	0.0%	-11.00 [-25.10, 3.10]
van Wagoner 2003	99	48	172	100	48	166	0.0%	-1.00 [-11.24, 9.24]
Donovan 2012	100	53	150	100	72	154	0.0%	0.00 [-14.19, 14.19]
Colby 2011	143	172	13	100	48	11	0.0%	43.00 [-54.71, 140.71]
**Subtotal (95% CI)**			**0**			**0**		**Not estimable**
Heterogeneity: Not applicable								
Test for overall effect: Not applicable								
**2.1.2 Oral vitamin C in non-US trials**								
Eslami 2007	92.37	46	50	100	49	50	5.3%	-7.63 [-26.26, 11.00]
Dehghani 2014	92.68	10	50	100	23	50	37.9%	-7.32 [-14.27, -0.37]
**Subtotal (95% CI)**			**100**			**100**	**43.1%**	**-7.36 [-13.87, -0.84]**
Heterogeneity: Chi² = 0.00, df = 1 (P = 0.98); I² = 0%								
Test for overall effect: Z = 2.21 (P = 0.03)								
**2.1.3 Intravenous vitamin C in non-US trials**								
Papoulidis 2011	80.61	61	85	100	61	85	5.4%	-19.39 [-37.73, -1.05]
Sarzaeem 2014	81.34	18	85	100	28	85	36.6%	-18.66 [-25.74, -11.58]
Sadeghpour 2015	84.75	49	113	100	49	177	13.7%	-15.25 [-26.81, -3.69]
Polymeropoulos 2015	88.53	42	11	100	52	11	1.2%	-11.47 [-50.97, 28.03]
Antonic 2016	106.8	60	52	100	60	53	0.0%	6.80 [-16.15, 29.75]
**Subtotal (95% CI)**			**294**			**358**	**56.9%**	**-17.76 [-23.43, -12.09]**
Heterogeneity: Chi² = 0.37, df = 3 (P = 0.95); I² = 0%								
Test for overall effect: Z = 6.14 (P < 0.00001)								
**Total (95% CI)**			**394**			**458**	**100.0%**	**-13.27 [-17.55, -8.99]**
Heterogeneity: Chi² = 5.94, df = 5 (P = 0.31); I² = 16%								
Test for overall effect: Z = 6.08 (P < 0.00001)								
Test for subgroup differences: Chi² = 5.57, df = 1 (P = 0.02), I² = 82.1%								

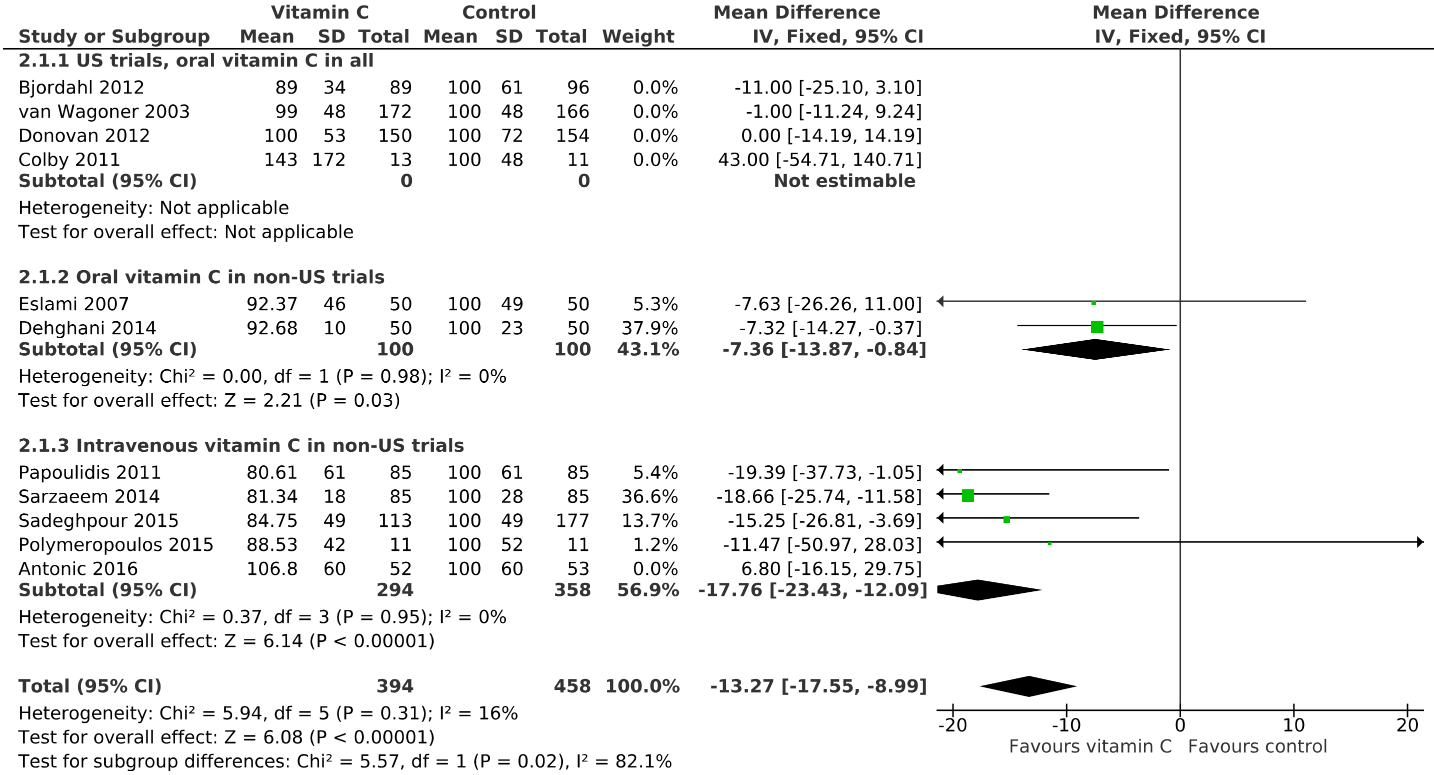

Supplement: Additional file 2: — Additional forest plots showing the calculations. (PDF 957 kb) [file 12872_2017_478_MOESM2_ESM.pdf]
